# Distributed Polarizability Model for Covalently Bonded Fullerene Nanoaggregates: Origins of Polarizability Exaltation

**DOI:** 10.3390/nano12244404

**Published:** 2022-12-09

**Authors:** Denis Sh. Sabirov, Alina A. Tukhbatullina

**Affiliations:** Laboratory of Mathematical Chemistry, Institute of Petrochemistry and Catalysis UFRC RAS, 450075 Ufa, Russia

**Keywords:** fullerene, fullerene dimer, fullerene trimer, isomerism, dipole polarizability, exaltation of polarizability, distributed polarizability

## Abstract

Polarizability exaltation is typical for (C_60_)*_n_* nanostructures. It relates to the ratio between the mean polarizabilities of (C_60_)*_n_* and C_60_: the first one is higher than the *n*-fold mean polarizability of the original fullerene. This phenomenon is used in the design of novel fullerene compounds and the understanding of its properties but still has no chemical rationalization. In the present work, we studied the distributed polarizability of (C_60_)_2_ and isomeric (C_60_)_3_ nanoaggregates with the density functional theory method. We found that polarizability exaltation increases with the size of the nanostructure and originates from the response of the sp^2^-hybridized carbon atoms to the external electric field. The highest contributions to the dipole polarizability of (C_60_)_2_ and (C_60_)_3_ come from the most remote atoms of the marginal fullerene cores. The sp^3^-hybridized carbon atoms of cyclobutane bridges negligibly contribute to the molecular property. A similar major contribution to the molecular polarizability from the marginal atoms is observed for related carbon nanostructures isomeric to (C_60_)_2_ (tubular fullerene and nanopeanut). Additionally, we discuss the analogy between the polarizability exaltation of covalently bonded (C_60_)*_n_* and the increase in the polarizability found in experiments on fullerene nanoclusters/films as compared with the isolated molecules.

## 1. Introduction

Fullerene multicage compounds are an interesting class of carbon-rich compounds, whose molecules contain two and more fullerene cores [1,2,3]. The cores may be directly connected via cyclobutane motifs resulting from the chemical reaction of [2+2] addition between the double bonds of the fullerene [4,5]. In the case of the C_60_ fullerene, this reaction occurs via the 6.6 bonds (this designation means that the bond is shared by two hexagons of the C_60_ skeleton). Thus, there is only one synthesized isomer of the [2+2] dimer (C_60_)_2_. The next addition of the fullerene cores is possible in different positions because in the (C_60_)_2_ dimer, the 6.6 bonds become inequivalent due to symmetry loss as compared with original C_60_. Such regioisomeric trimers were synthesized and studied experimentally and theoretically [6,7]. As found, molecular and physicochemical properties strongly depend on the addition pattern in fullerene trimers (C_60_)_3_. 

Covalently bonded C_60_ fullerene [2+2] aggregates are produced using the mechanochemical reaction induced by potassium/sodium cyanide or solid aromatic amines under the “high-speed vibration milling” conditions [2,4,6,8]. These ways lead to a fullerene dimer and trimers, which are large molecules visualized with scanning tunneling microscopy [6]. Indeed, these molecules are unusual due to their shape and size. They are rigid structures with no possible conformational transitions with distinct symmetries and topological properties [9]. This makes them potentially applicable in carbon nanotechnologies (e.g., for qubits [10,11] and molecular materials with nonlinear optic properties [12]). From a fundamental point of view, fullerene oligomers are simultaneously molecules and nano-objects. Unfortunately, this interesting feature creates some obstacles for chemical studies of these molecules, even for chemical identification. Fullerene oligomers larger than (C_60_)_3_ were detected only with mass spectrometry techniques that provide only the contents of the substances—not chemical structures [13,14].

As covalently bonded fullerene aggregates are promising for applications, there is a necessity to know their behavior in electric fields [15]. Previously, we systematically studied the dipole polarizability (hereinafter, polarizability) of various dimers, trimers, and higher oligomers of C_60_, C_70_, and other fullerenes. This quantity corresponds to the molecule’s response to the external electric field; it is applied in physical chemistry to assess reactivity, intermolecular interactions, optical properties, collisions, and so on [16]. To calculate the polarizability, we used density functional theory methods. The calculated values were interesting not only themselves—we found the superadditivity of the mean polarizabilities of (C_60_)*_n_* called exaltation of polarizability [3,5,7]. We observed the positive deviation of the calculated values α[(C_60_)*_n_*] from *n*α(C_60_). We were not stopped on the explanation of this phenomenon and focused on demonstrating that polarizability exaltation is typical for all classes of fullerene multicage compounds regardless of the chemical nature of the bridges between the fullerene units. After scrutinizing our works, Swart and van Duijnen [17] provided an explanation for the exaltation based on an analogy with the classical electric theory. They considered a fullerene dimer as a system of interacting dielectric spheres. This allowed for distinguishing parallel ($\alpha_{∥}$) and perpendicular components ($\alpha_{⊥}$) of mean polarizability (relative to the applied electric field) of such system, and these contributions could be estimated within the classic theory if key input parameters are extracted from the relevant quantum chemical calculations. In general, ($\alpha_{∥}$) and ($\alpha_{⊥}$) differ so that the exaltation arises. The model explains the polarizability exaltation for (C_60_)_2_ but works with assumptions in the case of higher oligomers (as the number of cages is greater, and we have shown that only the most remote cages define the exaltation value). Thus, there is still no chemical explanation for the polarizability exaltation of fullerene oligomers.

Recently, the distributed polarizability models have been applied in fullerene chemistry. This approach allows for assessing the contributions of the atoms to the molecular polarizability using the calculated atomic charges with and without an applied electric field [18]. In general, it is applicable to various chemical structures, from small molecules to proteins and nano-objects. As for fullerenes, this approach was applied to their endohedral complexes [19] and exohedral derivatives [20]. We believe that it could be useful for rationalizing the peculiarities of the dipole polarizability of fullerene oligomers. 

In the present work, we study the dipole polarizability of the fullerene dimer (C_60_)_2_ and trimers (C_60_)_3_ within the distributed polarizability model. Additionally, we discuss the relevance of our computed polarizability exaltation of (C_60_)*_n_* to experimental data and theoretical results of other groups.

## 2. Computational Details

The TPSS/TZVP density functional theory method was used for calculations (program Gaussian 09 Rev. D3.01 [21]). Our choice is based on our previous studies of fullerene and its derivatives whereby this method demonstrated high reliability in describing mean polarizabilities of the molecules. The structures of (C_60_)*_n_* and C_60_ itself as the reference compound were completely optimized. The confirmation that the obtained structures correspond to the minima of the potential energy surfaces was performed based on their hessian, which had no imaginary frequencies. The calculations of the dipole polarizability were performed according to the distributed polarizability technique [18]. In general, this methodology uses a finite-field approach, and its details are below. The optimized structures and output data for distributed polarizability calculations can be found in Supplementary Materials (Tables S1–S10).

The mean polarizabilities of the molecules are analytically calculated. To assess the atomic contributions to the molecular polarizability, the atomic partitions into the electronic density are calculated according to the Hirshfeld scheme [22]. The Hirshfeld atomic charges are computed in four modes for each structure: with fields *F_γ_* having one nonzero component along axis *γ* (*γ* = *x*, *y*, or *z*) and one calculation without the field. The *γ* component of the distributed dipole moment for atom *i* for each field *F_γ_* is calculated as

(1) $$\mu_{\gamma i}=q_{i}R_{\gamma i}+\delta \mu_{\gamma i}$$

where *q_i_* and *R_γi_* are the Hirshfeld atomic charges and Cartesian coordinates of the atoms. The δ*μ_γi_* values are taken as is from the Gaussian output file in a.u. The *γγ* component of the contribution of the *i*-th atom to the mean polarizability of the molecule follows from the definition of polarizability (below, ***μ*_ind_** is the induced dipole moment):

(2) $$\mu_{ind}=\alpha F$$

and equals

(3) $$\alpha_{\gamma \gamma i}=\frac{\mu_{\gamma i}\left(F_{\gamma }\right)-\mu_{\gamma i}\left(0\right)}{F_{\gamma }}$$

where *F_γ_* = −0.001 a.u. (the recommended value [19]). The isotropic value corresponding to atom *i* equals:

(4) $$\alpha_{i}=\frac{1}{3}\left(\alpha_{xxi}+\alpha_{yyi}+\alpha_{zzi}\right)$$

The summation of atomic values *α_i_* for all atoms of the nanostructure gives its molecular polarizability:

(5) $$\alpha_{{\left(C_{60}\right)}_{n}}=\overset{60n}{\underset{i=1}{\sum }}\alpha_{i}$$

The polarizability exaltation of (C_60_)*_n_* is calculated as the difference between the actual molecular polarizability (obtained according to Equation (5)) and the corresponding additive value. The last one is based on the proposition that the polarizability of (C_60_)*_n_* results from *n* molecular polarizabilities of original C_60_ (this reference value equals 80.3 Å^3^ as calculated with the TPSS/TZVP method):

(6) $$\Delta \alpha_{{\left(C_{60}\right)}_{n}}=\alpha_{{\left(C_{60}\right)}_{n}}-n\alpha_{C_{60}}$$

## 3. Results

**Figure 1:**
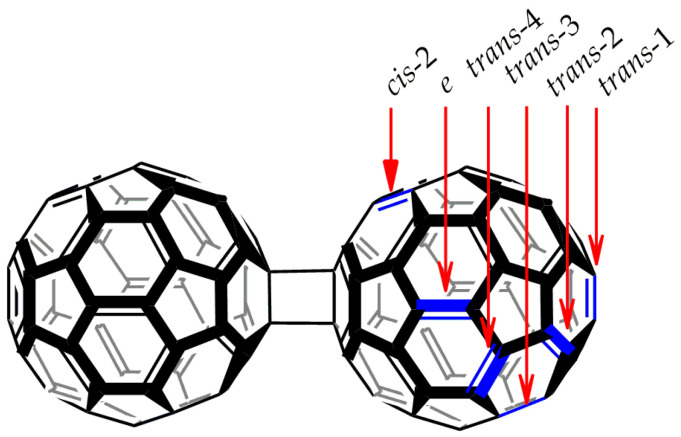
The formula of a [2+2] fullerene dimer (C_60_)_2_. The 6.6 bonds, which are able to be connected to the other C_60_ core, are shown with arrows. Their designation corresponds to the conventional nomenclature of the C_60_ bisadducts [23].

The general formula of the fullerene oligomers studied in the present work is shown in Figure 1. In this study, we focus on the experimentally obtained (C_60_)_2_ and (C_60_)_3_ structures. For designations of trimers, we use rational nomenclature designations to specify the addition patterns (Figure 1). Note that in the (C_60_)_2_ molecule, there are nine nonequivalent 6.6 bonds, which are able to attach chemical groups (*cis*-1, *cis*-2, *cis*-3, *ee*, *ef*, *trans*-4, *trans*-3, *trans*-2, and *trans*-1). As C_60_ is a bulky molecular fragment, only six positions are open for attaching the next C_60_ core to (C_60_)_2_. These are *cis*-2, *ee*, *ef*, *trans*-4, *trans*-3, *trans*-2, and *trans*-1 [23]. Herewith, the addition to the equatorial bonds *ee* and *ef* leads to the same structure designated as *e*-(C_60_)_3_. In the case of the *cis*-2 addition, the marginal C_60_ cores are arranged too close so that they are bridged (also via a *cis*-2 pattern). This trimer is called cyclic, and structurally, it stays apart from the other (C_60_)_3_, which have the central and marginal fullerene cores. Figure 1 shows only the bonds taking part in the addition reactions resulting in the fullerene trimers. All of the discussed structures were synthesized and separated as individual substances [2,4,6,8] except for *trans*-1-(C_60_)_3_. The latter structure is least stable among (C_60_)_3_. However, there is an experimental detection of the *trans*-1 addition pattern in (C_60_)*_n_* polymers obtained inside the carbon nanotubes [24]. 

The optimized geometries of the fullerene compounds are collected in Supplementary Materials. The TPSS/TZVP-calculated values on (C_60_)_2_ and (C_60_)_3_ polarizability are shown in Table 1. According to the additive scheme (Equation (6)), the polarizability exaltation is typical for all (C_60_)_3_. It means that the mean polarizabilities of the (C_60_)*_n_* molecules are higher than the *n*-fold mean polarizability of the original fullerene (*n* = 2 or 3). These computational results agree with the previous estimates with the PBE/3ζ method [5,7]. 

Table 1 also contains the results of the distributed polarizability calculations, that is, contributions to the molecular mean polarizability from the central and marginal cores. These values have been obtained quantum-chemically as the sums of the atomic contributions to α[(C_60_)*_n_*]. The atomic contributions themselves are collected in Supplementary Materials and visualized as the polarizability maps in Section 4.2. In this section, the ball-and-cylinder models of the nanostructures under study could be found.

## 4. Discussion

### 4.1. Relevance to the Experimental Data on the C_60_ Polarizability

We previously reviewed experimental and theoretical works on fullerene polarizability (see, e.g., our work [7] and some key experimental works in this field [25,26,27,28,29,30]). Based on that analysis, the mean polarizability of C_60_ is approximately 80 Å^3^, so our computed value of 80.3 Å^3^ perfectly agrees. As for the (C_60_)_2_ and (C_60_)_3_ molecules, there are no experimental data on their dipole polarizabilities. We consider that the absence of the experiments is due to the facile dissociation of such fullerene compounds under optical treatments required for typical polarizability measurements. Nevertheless, we would like to discuss the relevance of the computed polarizability exaltation to the experimental *α*(C_60_) values deduced from the experiments on C_60_ in different states.

Munn and Petelenz [31] found that the measured α(C_60_) values increase from the isolated molecules to solid: ~76.5 Å^3^ for the molecules in the gas phase < ~79 Å^3^ for fullerene clusters containing several C_60_ molecules < ~85 Å^3^ for the fullerene films. Usually, such a mismatch between the polarizability of the isolated molecules α and the effective molecular polarizability α_eff_ deduced from solid-state measurements is due to (i) the changes in molecular structure under the transit to the condensed state, (ii) compression of electronic clouds of the molecules, (iii) inducing heterogeneous electric fields in the formed crystals, and so on [32]. All mentioned points are not applicable to fullerene: as is known, (i) C_60_ keeps the initial molecular geometry in a crystal (fullerite), (ii) compressing electronic clouds must decrease the resulting polarizability, and (iii) the emergence of heterogeneous electric fields in fullerite has low probability due to the high symmetry of the molecule. The authors [31] explained the mentioned increase with the generation of the instantaneous charges on the fullerene cages in neighboring crystal lattice nodes and provided a relation for assessing the polarizability exaltation effect. Their model indicates the possibility of 30% of polarizability gain in crystals as compared with the isolated C_60_ molecules. Note that the instantaneous charges invoked for explaining aggregate-state-dependent polarizability exaltation of C_60_ are not observables.

In our calculations (Table 1), the mean polarizabilities of C_60_ moiety deduced from the calculated (C_60_)*_n_* are ~9…15% higher than the computed α(C_60_) (the range of percentages corresponds to (C_60_)_2_…*trans*-1-(C_60_)_3_). This increase corresponds to the observed one for fullerene samples. However, we compare similar but not the same cases. In this work, we deal with the chemically bonded C_60_ cores. Thus, we hypothesize that the polarizability exaltation of (C_60_)_2_ and higher chemically bonded fullerene oligomers is analogous to increasing the mean polarizability of C_60_ from gas to crystalline state. We assume that the nature of both polarizability exaltations relates to interacting π-electron systems of the C_60_ cores regardless of whether they are bonded with chemical bonds or intermolecular forces. This hypothesis will be tested with relevant computational techniques in our further works.

**Figure 2:**
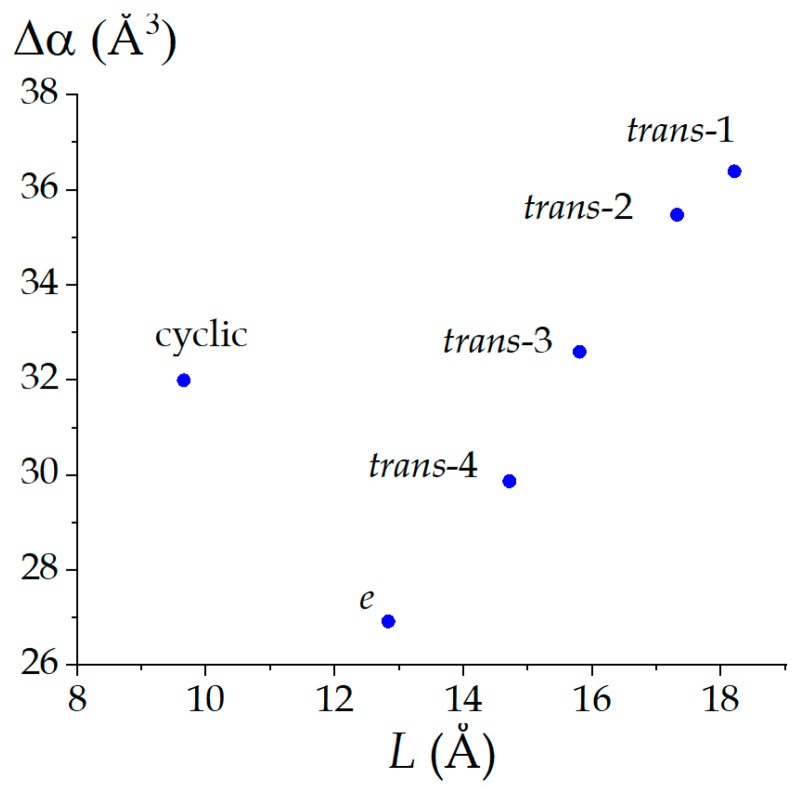
Dependence of the polarizability exaltation on the distance between the marginal fullerene cores in (C_60_)_3_. The data associated with the plot could be found in Table 1.

Previously, we studied the influence of molecular topology and molecular geometry on the mean polarizability of isomeric (C_60_)_6_ [33]. As found, the geometric factor has a major effect on α: the resulting α[(C_60_)_6_] values increase with the maximal remoteness of the C_60_ cores in the nanostructure. The DFT computations of the present study fit into this regularity: we demonstrate it with the polarizability exaltation values (Figure 2). However, the molecular topology also has an effect. This follows from the mean polarizability of the cyclic trimer, which falls out the regularity. Topologically [9], this trimer stays apart from other (C_60_)_3_ due to its closed structure. 

Note that Δ*α* values relate to a rough estimation of the polarizability effects of (C_60_)*_n_*. They are based on the assumption that all fullerene cores equally contribute to the molecular property, but this holds true only for cyclic (C_60_)_3_. To look deeper into the matter of the fullerene nanostructures, we apply the distributed polarizability model below. 

### 4.2. Distributed Polarizability Model of (C_60_)_3_: The Responses of the Cores Differ

In the distributed polarizability computations, we obtained the atomic contributions to the molecular mean polarizabilities of the (C_60_)_3_ trimers. These data allow for assessing the contributions of the fullerene cores to the molecular property (*α*). We found that, in general, the contribution of the central core α_centr_ (here, we do not treat (C_60_)_2_ and cyclic (C_60_)_3_, that is, the molecules, whereby the cores are identical) is pronouncedly lower as compared with the marginal ones (Table 1). Only in the most compact nanoaggregates cyclic and *e*-(C_60_)_3_, α_centr_ > α(C_60_). Other structures demonstrate diminishing contributions α_centr_ relative to the mean polarizability of the original fullerene, α(C_60_). In the case of the marginal fullerene cores, α_marg_ > α(C_60_) for all studied nanostructures (including (C_60_)_2_, which could be considered to be constructed with two marginal cores), and this enlargement varies in the range of 6.97…24.73 Å^3^. 

**Figure 3:**
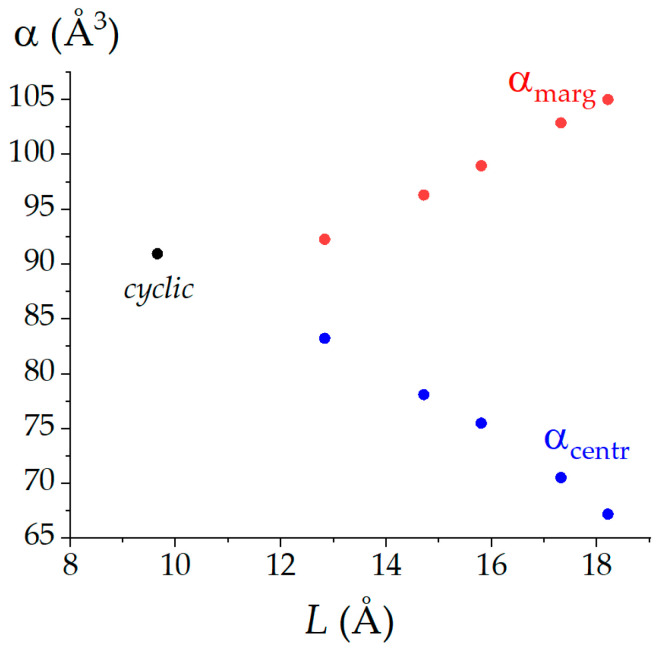
The contributions from central and marginal fullerene cores (α_centr_ and α_marg_, respectively) to the mean polarizabilities of the (C_60_)_3_ nanostructures as a function of the marginal cores’ remoteness. The point corresponding to the cyclic fullerene trimer is separately selected as all its fullerene units are equivalent. The data associated with the plot can be found in Table 1.

It is obvious that the mentioned effects in (C_60_)_3_ depend on the shape of the nanostructures. A decrease in the polarizability contributions of the central cores and an increase in the polarizability contributions of the marginal ones become greater with the distance between the last ones (Figure 3). Accordingly, the marginal cores play a crucial role in the polarizability exaltation effects as their contributions dominate.

There is an analogy between the fullerene derivatives that belong to different classes. A similar situation is observed in the case of fullerene bisadducts C_60_X_2_ (X = O and CH_2_) [20]: the contribution of the C_60_ moiety in these compounds goes down when increasing the distance between X. In our case, the peripheral fullerene cores play the role of addends X (i.e., X = C_60_).

### 4.3. Distributed Polarizability Maps of (C_60_)_3_

**Figure 4:**
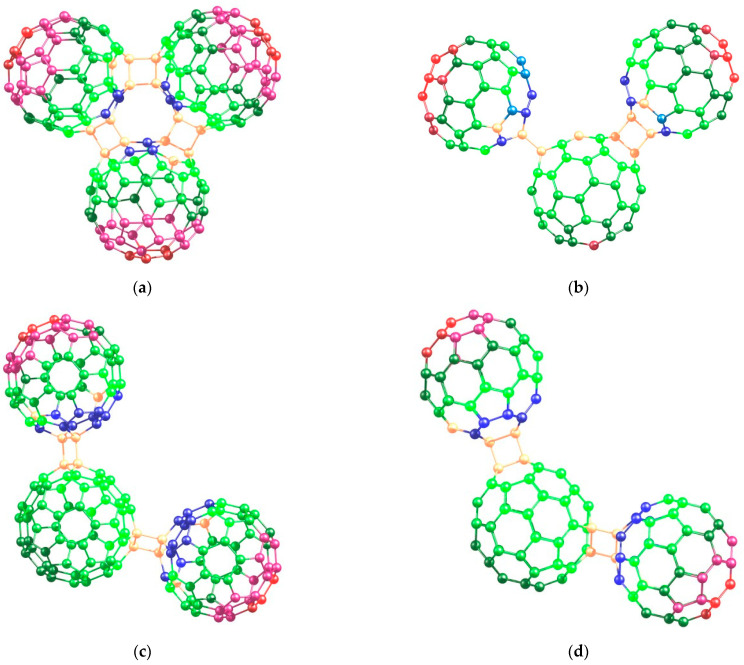

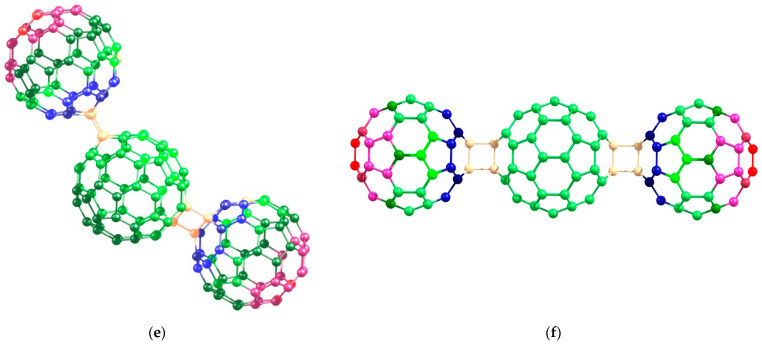
The distributed polarizability maps of the (C_60_)_3_ trimers: (**a**) cyclic, (**b**) *e*, (**c**) *trans*-4, (**d**) *trans*-3, (**e**) *trans*-2, and (**f**) *trans*-1. Color code: blue—negative atomic contributions; other colors—positive atomic contributions: beige (0%–4%), green (5%–68%), pink (69%–96%), and red (96%–100%) correspond to the percentage of the contribution relative to the maximal contribution to the dipole polarizability of the nanostructure.

At the last stage of detailing the polarizability of fullerene covalently bonded nanoaggregates, we analyze the atomic contributions themselves. The corresponding numerical values are presented in Supplementary Materials. Here, it is convenient to visualize them as the colored maps (Figure 4). 

The maps were drawn from the following considerations. To reveal regularities general for all (C_60_)_3_, we selected in each nanostructure the atoms with the highest contributions and took them as 100%. Other atoms, which have lower contributions, were discriminated depending on the percentage relative to the highest contribution in the structure (Figure 4). We must work with percentages because maximal contributions are not the same in different (C_60_)_3_.

According to the performed computations, the most polarizable atoms in (C_60_)_3_ are located at the margins of the marginal fullerene cores (Figure 4, red atoms). It means that the atomic charges are induced at these atoms when the nanostructure interacts with the electric field. When moving to the central cores of (C_60_)_3_, the atomic contributions to the molecular property become lower. The fact that the maximal charges should arise on the most distant atoms allows for rationalizing the size dependence of the polarizability exaltation of (C_60_)_3_. Indeed, any dipole moment (both permanent and induced as in Equation (2)) depends on the distance ***L*** between the centers of the opposite-sign charges in the molecule:

(7) $$\mu_{ind}=\Delta qL$$

where **Δ*q*** is the charge transfer value [34]. Thus, according to the distributed polarizability calculations, the charges emerge on the most distant atoms of (C_60_)_3_. This is reflected with the polarizability exaltation effect increasing with the linear size of the nanostructure (and the remoteness of the marginal fullerene cores plays a parameter of the size).

Further, we pay attention to the atoms with the smallest positive atomic contributions to the molecular polarizability of (C_60_)_3_ (Figure 4, beige atoms). These atoms are mainly sp^3^-hybridized atoms of the cyclobutane moieties that connect fullerene units in one nanostructure. We were surprised to find in the studied nanostructures the atoms with negative atomic contributions. Indeed, dipole polarizability is always a positive value. However, this molecular property may be “nonuniformly” distributed over the atoms. In the classic theory of chemical structure, polarizability is associated with the volume of the molecule’s electronic cloud (in favor of it, α has the dimension of volume in centimeter–gram–second system of units). We believe that the negative atomic contributions relate to the shift of the electronic density to the other moieties of the molecule. Anyway, the atoms with nonzero contributions are sp^2^-hybridized, and their contributions relate to the response of the rich π-electron systems of the fullerene cores. Thus, atoms of these types are responsible for the polarizability exaltation of (C_60_)*_n_*. In contrast, the site of the connection of the fullerene units has no π-electrons, and therefore, their contributions are negligible. A similar situation is observed for other chemical objects. For example, the dipole polarizability of nanodiamonds [35] consisting of only sp^3^-hybridized carbon atoms linearly increases with the size of the particle; that is, there is no polarizability exaltation in sp^3^-carbon nanostructures. 

### 4.4. Prospective: From Fundamental Properties to Nanoapplications

The polarizability exaltation of the fullerene dimer and oligomers has been known for almost 10 years and studied with quantum-chemical and classical approaches [5,7,17]. The present computational study allows for deeper understanding this phenomenon in fundamental and applied aspects.

**Figure 5:**
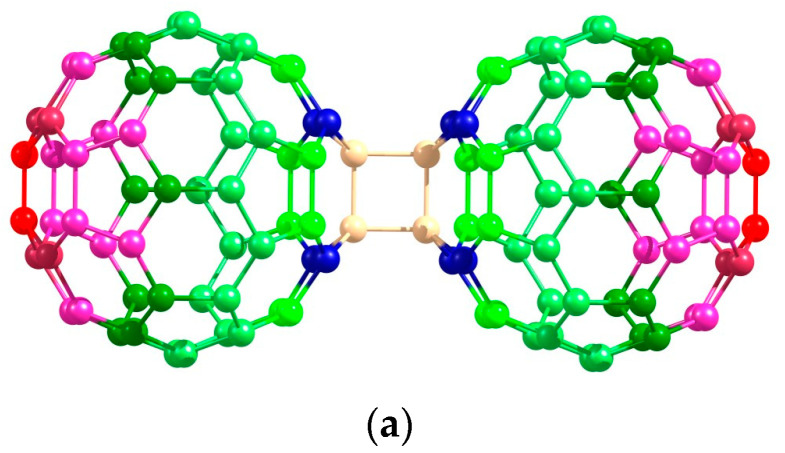

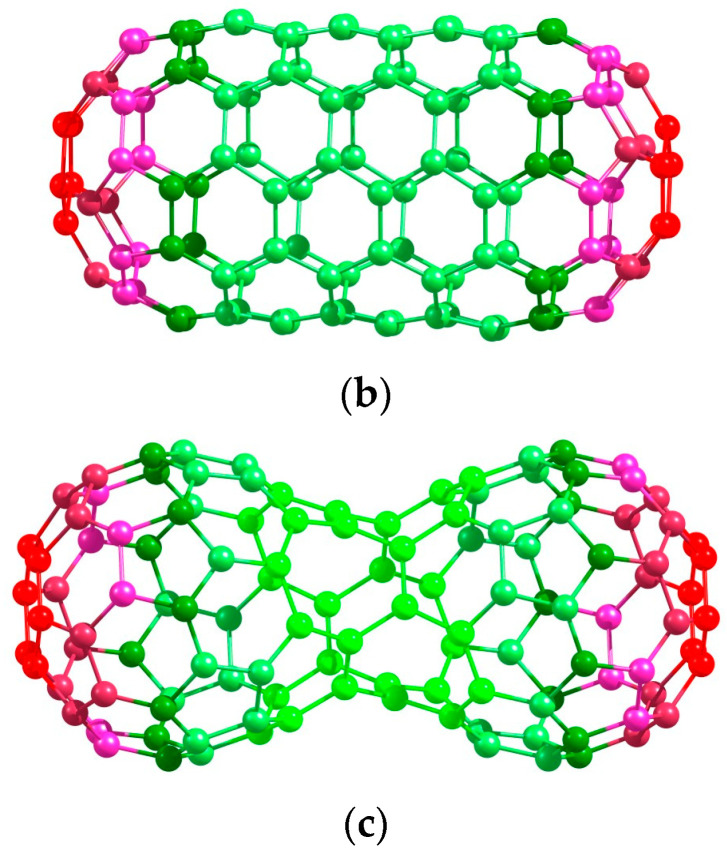
The distributed polarizability maps of the (C_60_)_2_ dimer (**a**) and its isomers, tubular fullerene (**b**), and nanopeanut (**c**). The color code is the same with Figure 4.

For this purpose, we compare three isomeric all-carbon nano-objects: [2 + 2] dimer, tubular fullerene, and nanopeanut (Figure 5). The two last structures represent convenient model objects for structural studies [9,36,37,38,39]. Their distributed polarizability maps have been obtained similarly to the title compounds. Interestingly, the marginal atoms of the two novel all-carbon objects also provide the major contributions to the molecular polarizability. Herewith, all three structures differ: the structure of the fullerene dimer has been discussed above; the C_120_ fullerene consists of the hexagons and isolated pentagons since a nanopeanut’s structures include heptagons in the fusion motif. In all cases, marginal carbon atoms make decisive contributions to the molecular polarizability, and it seems to be a common property of carbon nanostructures. Central atoms of these nanostructures have smaller contributions. 

This theoretical study is a part of our ongoing research program on the polarizability of fullerene compounds. Besides the fundamental interest in the relations between their chemical structure and polarizability parameters [40], it has the applied aspects. For example, dipole polarizability plays a crucial role in assessing the collisions of fullerene nanoclusters in the gas phase [41] and their ordering in films [42,43]. As recently found, fullerene dimers demonstrate catalytic properties due to the polarizability exaltation [44,45]. As for nanodevices, there are examples of thermally driven fullerene-based nanocars [46], but their analogues, which could be manipulated with electric fields [47], are a matter of the future. Here, dipole polarizability and its exaltation could provide novel insights into the design of the fullerene-based nanostructures with a high response to external electric fields. Dipole polarizability estimates have already been used in the synthetic chemistry of fullerene-based materials from C_60_ and C_70_ under the action of various fields [48,49,50,51].

## 5. Conclusions

The polarizability exaltation of (C_60_)*_n_* is a phenomenon that consists of the relation α[(C_60_)*_n_*] > α(C_60_). Our quantum-chemical computations within the distributed polarizability model allows for assessing the atomic contributions to the molecular polarizability of (C_60_)*_n_*. Using (C_60_)_3_ as the simplest example, we have shown that the polarizability exaltation of covalently bonded C_60_ nanoaggregates originates from the response of the sp^2^-hybridized carbon atoms to the external electric field. Atoms of this type are the centers, where facilely polarizable π-electrons are localized. The biggest contributions to the dipole polarizability of (C_60_)_3_ come from the most remote atoms of the marginal fullerene cores. This explains why the polarizability effect increases with the remoteness of the marginal fullerene cores in (C_60_)_3_. 

We point the analogy between the polarizability exaltation of covalently bonded (C_60_)*_n_* and the increase in the polarizability found in experimental studies of fullerene nanoclusters and solids as compared with the isolated molecules. Indeed, both carbon nanomaterials contain rich π-electron systems. The difference is in the presence or absence of the covalent bonds between the fullerene cores in the mentioned structures. As our computations reveal that the π-electron systems play a decisive role for the polarizability exaltation, we assume the common nature of these two phenomena.

The major contributions of marginal atoms to the molecular polarizability is also typical for other carbon nanostructures (tubular fullerene and nanopeanut). It seems that oblong carbon nanostructures share this feature. We believe that it could be useful in material science and nanotechnology. 

## Figures and Tables

**Table 1:** The TPSS/TZVP-calculated polarizability and structural parameters of (C_60_)_2_ and (C_60_)_3_.

Molecule	Mean Polarizability, α (Å^3^)	Polarizability Exaltation, Δα (Å^3^) ^1^	Central Core Contribution (Å^3^)	Marginal Core Contribution (Å^3^)	Remoteness of Marginal Cores, *L* (Å) ^2^
(C_60_)_2_	174.54	13.94	n/a	87.27	9.710
Cyclic (C_60_)_3_	272.90	32.00	90.97 ^3^	90.97 ^3^	9.655
*e*-(C_60_)_3_	267.83	26.93	83.26	92.28	12.833
*trans*-4-(C_60_)_3_	270.77	29.87	78.13	96.32	14.714
*trans*-3-(C_60_)_3_	273.49	32.60	75.52	98.99	15.804
*trans*-2-(C_60_)_3_	276.38	35.48	70.56	102.91	17.319
*trans*-1-(C_60_)_3_	277.29	36.39	67.22	105.03	18.210

^1^ Calculated with Equation (6), taking the TPSS/TZVP-calculated mean polarizability of the C_60_ fullerene equal to 80.3 Å^3^. ^2^ Remoteness means the distance between the mass centers of the fullerene cores. Note that all distances between the fullerene cages are the same in cyclic (C_60_)_3_. ^3^ No marginal and central cores could be selected in cyclic (C_60_)_3_ due to its closed structure. All fullerene cores contribute equally to the molecular property.

## Data Availability

Not applicable.

## Supplementary Materials

The following supporting information can be downloaded at: https://www.mdpi.com/article/10.3390/nano12244404/s1, Tables S1–S10: Cartesian coordinates of the carbon nanostructures; Tables S11 and S12: Atomic contributions to the mean polarizability of C_60_, (C_60_)_2_, (C_60_)_3_, and related nanostructures C_120_.
