# Supplementary material for: Distributed Polarizability Model for Covalently Bonded Fullerene Nanoaggregates: Origins of Polarizability Exaltation
Source: Nanomaterials (Basel). 2022 Dec 9;12(24):4404. doi: 10.3390/nano12244404 (PMC9781774; doi:10.3390/nano12244404)
Supplement: Supplementary file 1 [file nanomaterials-12-04404-s001.zip › nanomaterials-2087186-supplementary.pdf]

Supplementary Materials

to the article

Distributed Polarizability Model for Covalently-Bonded Fullerene Nanoaggregates: Origins of Polarizability Exaltation

Denis Sh. Sabirov [1,*], Alina A. Tukhbatullina [1]

[1] Laboratory of Mathematical Chemistry, Institute of Petrochemistry and Catalysis UFRC RAS; ink@anrb.ru

* Correspondence: diozno@mail.ru; Tel.: +7 (347) 284 27 50

Table S1. Cartesian coordinates of C_{60} (hereinafter, in angstroms)

2.869482	2.084958	-0.161739
1.848463	3.031423	-0.039038
3.194591	1.218253	0.957745
1.112204	3.148000	1.207773
2.486219	1.330519	2.157332
1.424719	2.313820	2.284639
2.997153	1.308530	-1.382756
0.914857	3.238762	-1.132689
2.098775	1.507890	-2.434721
1.037610	2.491589	-2.307376
-0.276324	3.426904	0.884637
-0.398401	3.483129	-0.561801
-1.298912	2.861508	1.650915
-1.538702	2.971088	-1.187206
-2.484322	2.330302	1.000811
-2.601839	2.383686	-0.390740
-2.892210	1.134852	1.718403
-3.131638	1.244078	-1.119402
-3.401759	0.038241	1.017768
-3.523619	0.093955	-0.428765
-2.997153	-1.308530	1.382756
-3.194591	-1.218253	-0.957745
-2.869482	-2.084958	0.161739
3.523619	-0.093955	0.428765
3.401759	-0.038241	-1.017768
3.131638	-1.244078	1.119402
2.892210	-1.134852	-1.718403
2.601839	-2.383686	0.390740
2.484322	-2.330302	-1.000811
1.538702	-2.971088	1.187206
1.298912	-2.861508	-1.650915
0.398401	-3.483129	0.561801
0.276324	-3.426904	-0.884637
-0.914857	-3.238762	1.132689
-1.112204	-3.148000	-1.207773
-1.848463	-3.031423	0.039038
-2.098775	-1.507890	2.434721
-1.037610	-2.491589	2.307376
-1.570043	-0.368014	3.163613
0.147607	-1.959120	2.957035
-0.181444	-0.646925	3.486360
0.765693	0.379997	3.446395
2.079088	0.135243	2.875420
2.395784	-1.127316	2.366549

-1.958897	0.927551	2.812075
0.361556	1.726615	3.081663
-0.974397	1.994926	2.770776
1.410830	-2.194337	2.407801
-1.424719	-2.313820	-2.284639
-2.486219	-1.330519	-2.157332
-0.361556	-1.726615	-3.081663
-2.079088	-0.135243	-2.875420
-0.765693	-0.379997	-3.446395
0.181444	0.646925	-3.486360
1.570043	0.368014	-3.163613
1.958897	-0.927551	-2.812075
0.974397	-1.994926	-2.770776
-0.147607	1.959120	-2.957035
-1.410830	2.194337	-2.407801
-2.395784	1.127316	-2.366549

Table S2. Cartesian coordinates of [2+2]-$(C_{60})_2$

-5.282303	2.312907	2.598768
-5.737027	3.041139	1.425645
-6.866704	2.604683	0.726672
-7.587023	1.426989	1.174626
-7.150272	0.727990	2.304741
-5.977438	1.176856	3.026551
-3.832132	2.315485	2.592072
-3.390657	3.040085	1.425440
-4.564654	3.492288	0.698037
-4.564682	3.492232	-0.698404
-6.866732	2.604604	-0.726875
-8.033364	0.699105	0.000022
-8.033371	-0.699080	0.000094
-7.587038	-1.426841	1.174778
-7.150281	-0.727726	2.304819
-5.977450	-1.176529	3.026677
-5.246419	0.000183	3.467038
-3.850052	0.000177	3.445662
-3.125653	1.176123	3.000810
-2.263263	2.590385	0.725549
-1.511878	1.450424	1.176156
-1.951748	0.740292	2.276862
-1.951750	-0.740081	2.276948
-3.125661	-1.175823	3.000941
-3.832155	-2.315224	2.592328
-5.282330	-2.312633	2.599016
-5.737052	-3.040979	1.425963

-6.866727	-2.604587	0.726948
-7.587060	1.426862	-1.174676
-5.282414	-2.312912	-2.598754
-5.977549	-1.176854	-3.026512
-5.246531	-0.000178	-3.467012
-3.850165	-0.000166	-3.445697
-3.125765	-1.176129	-3.000879
-3.390732	-3.040081	-1.425486
-4.564701	-3.492278	-0.698043
-5.737100	-3.041129	-1.425612
-6.866748	-2.604661	-0.726600
-7.587072	-1.426965	-1.174526
-7.150346	-0.727972	-2.304655
-5.977543	1.176541	-3.026629
-5.282395	2.312634	-2.598986
-3.832209	2.315221	-2.592337
-3.125746	1.175812	-3.000934
-1.951821	0.739981	-2.276954
-1.951842	-0.740330	-2.276970
-1.511935	-1.450465	-1.176250
-2.263301	-2.590423	-0.725625
-4.564680	-3.492209	0.698399
-3.390692	-3.039932	1.425762
-2.263293	-2.590343	0.725825
-1.511912	-1.450328	1.176298
-0.798600	-0.800507	-0.000025
-0.798572	0.800486	-0.000121
-1.511904	1.450352	-1.176433
-2.263289	2.590352	-0.725903
-3.390713	3.039946	-1.425790
-7.150346	0.727746	-2.304731
-5.737076	3.040990	-1.425928
-3.832233	-2.315503	-2.592122
7.587037	-1.426779	1.174850
8.033373	-0.699075	0.000132
8.033358	0.699110	-0.000016
7.587016	1.427051	1.174552
7.150260	0.728108	2.304698
7.150275	-0.727607	2.304854
6.866731	-2.604550	0.727082
6.866757	-2.604702	-0.726467
7.587075	-1.427019	-1.174451
7.150353	-0.728088	-2.304618
7.587059	1.426809	-1.174749
6.866699	2.604724	0.726542
5.737022	3.041217	1.425493

5.282301	2.313047	2.598658
5.977432	1.177013	3.026491
5.246414	0.000360	3.467039
5.977449	-1.176375	3.026737
5.282327	-2.312502	2.599133
5.737059	-3.040909	1.426123
5.737105	-3.041199	-1.425453
4.564711	-3.492319	-0.697862
4.564691	-3.492177	0.698576
3.390695	-3.039856	1.425906
3.832156	-2.315091	2.592443
3.125653	-1.175671	3.000990
3.850045	0.000354	3.445664
3.125645	1.176282	3.000765
3.832123	2.315630	2.591974
6.866726	2.604576	-0.727005
2.263289	2.590268	-0.726031
3.390712	3.039843	-1.425948
3.832215	2.315085	-2.592466
3.125746	1.175653	-3.001038
1.951806	0.739938	-2.277079
0.798594	0.800474	-0.000131
1.511898	1.450486	1.176084
2.263279	2.590420	0.725418
3.390654	3.040162	1.425297
4.564648	3.492322	0.697862
4.564674	3.492184	-0.698579
5.282388	2.312506	-2.599108
5.977541	1.176390	-3.026689
5.246529	-0.000347	-3.467010
3.850163	-0.000363	-3.445687
3.125757	-1.176295	-3.000808
1.951820	-0.740445	-2.276928
1.511948	-1.450523	-1.176181
0.798599	-0.800520	0.000008
1.951742	0.740416	2.276846
1.951741	-0.739961	2.276991
1.511894	-1.450263	1.176376
2.263295	-2.590295	0.725960
2.263323	-2.590450	-0.725493
3.390742	-3.040160	-1.425331
3.832243	-2.315632	-2.591993
5.282419	-2.313038	-2.598635
5.977549	-1.176996	-3.026439
5.737070	3.040922	-1.426081
7.150350	0.727631	-2.304770

1.511927	1.450228	-1.176475

Table S3. Cartesian coordinates of tubular fullerene C_{120}

4.287632	2.611580	-2.258730
4.287504	1.459599	-3.128746
3.083204	0.791055	-3.414215
1.847867	2.613654	-2.271607
1.847820	1.471608	-3.133833
0.613118	0.783685	-3.358458
-0.613103	2.608840	-2.256837
-0.613063	1.458763	-3.125169
-1.847774	0.778972	-3.373315
-3.083302	2.647290	-2.297419
-3.083190	1.487347	-3.173614
-4.287440	0.785067	-3.362018
-5.524561	2.328539	-1.925896
-5.524450	1.216855	-2.765717
-4.287664	3.020422	-1.673508
-1.847845	3.033547	-1.670820
0.613132	3.018089	-1.671367
3.083371	3.069315	-1.693611
5.524560	2.610126	-1.522332
6.301928	1.458333	-1.929982
5.524465	0.751562	-2.926561
5.524557	-0.641424	-2.952878
3.083229	-0.662355	-3.441669
0.613081	-0.657151	-3.385832
-1.847787	-0.651779	-3.400424
-4.287452	-0.658282	-3.389204
-5.524439	-1.112118	-2.809431
-6.301948	0.045427	-2.418711
4.287585	-1.341349	-3.181719
1.847813	-1.353080	-3.187231
-0.613120	-1.340532	-3.177946
-3.083213	-1.367141	-3.227180
-5.524573	-2.254552	-2.011924
-3.083300	-2.559060	-2.395003
-0.613125	-2.522217	-2.353038
1.847847	-2.526386	-2.367984
4.287606	-2.524938	-2.355140
3.083328	-3.003235	-1.807449
0.613103	-2.952975	-1.783341
-1.847889	-2.968406	-1.783274
-4.287659	-2.955379	-1.785588
5.524550	-2.551222	-1.619259
5.524580	-3.006573	-0.302574

4.287710	-3.440735	0.292397
3.083380	-3.478554	-0.433679
0.613125	-3.424241	-0.421478
-1.847922	-3.436450	-0.430992
-4.287734	-3.427345	-0.421300
-3.083426	-3.492579	0.302877
-0.613137	-3.437769	0.292707
1.847873	-3.450256	0.301777
4.287665	-3.020410	1.673501
1.847849	-3.033521	1.670803
-0.613141	-3.018088	1.671369
-3.083382	-3.069325	1.693623
-5.524655	-3.015892	0.189506
-5.524563	-2.610118	1.522333
-4.287640	-2.611571	2.258729
-1.847877	-2.613641	2.271599
0.613101	-2.608870	2.256854
3.083306	-2.647304	2.297432
5.524566	-2.328541	1.925892
5.524451	-1.216853	2.765708
4.287445	-0.785072	3.362014
3.083196	-1.487347	3.173602
1.847773	-0.778981	3.373358
0.613084	-1.458763	3.125123
-1.847823	-1.471607	3.133841
-4.287508	-1.459591	3.128748
-0.613112	-0.783700	3.358479
-3.083207	-0.791045	3.414209
-6.302118	-2.286520	-0.790650
6.301964	-1.384989	-1.983414
6.302018	-2.314445	0.704255
4.287452	0.658274	3.389186
1.847797	0.651773	3.400375
-0.613101	0.657121	3.385814
-3.083236	0.662365	3.441681
-5.524468	-0.751554	2.926559
-5.524561	0.641432	2.952884
-4.287591	1.341349	3.181721
-1.847809	1.353086	3.187225
0.613099	1.340527	3.178004
3.083212	1.367150	3.227190
5.524443	1.112120	2.809437
5.524568	2.254549	2.011921
3.083306	2.559055	2.394993
0.613141	2.522149	2.353014
-1.847838	2.526390	2.367979
-4.287609	2.524954	2.355153
-3.083325	3.003221	1.807443

-0.613093	2.952964	1.783329
1.847875	2.968443	1.783303
4.287662	2.955391	1.785599
6.301952	-0.045424	2.418711
-6.301936	-1.458327	1.929986
5.524656	3.015901	-0.189505
3.083420	3.492587	-0.302866
0.613137	3.437787	-0.292708
-1.847870	3.450229	-0.301778
-4.287706	3.440727	-0.292397
4.287735	3.427354	0.421305
1.847916	3.436419	0.430998
-0.613122	3.424244	0.421481
-3.083375	3.478559	0.433681
7.048611	0.744834	-0.985631
7.048500	-0.707296	-1.012857
7.048588	-1.181964	0.359725
7.048665	-0.023151	1.235331
-6.302013	2.314445	-0.704259
-5.524576	3.006575	0.302572
-6.301962	1.384995	1.983410
-5.524545	2.551226	1.619258
-7.048500	0.707303	1.012855
-7.048609	-0.744826	0.985628
-7.048696	-1.167752	-0.403840
-7.048665	0.023155	-1.235334
-7.048583	1.181967	-0.359729
7.048693	1.167755	0.403838
6.302117	2.286525	0.790648

Table S4. Cartesian coordinates of nanopeanut C_{120}

-7.574736	-0.561011	1.312410
-7.574693	0.882228	1.122130
-6.740919	1.471908	2.151601
-6.234905	0.393336	2.983351
-6.740881	-0.863867	2.459452
-5.918216	-1.998270	2.445822
-4.932582	0.457342	3.469615
-7.574738	-1.412804	0.202766
-7.574703	1.417328	-0.170359
-5.918164	2.564010	1.844522
-6.740973	-2.599220	0.198797
-5.918245	-2.879292	1.298116
-4.554205	-1.916646	2.933794
-4.086197	-0.716565	3.450837
-4.086119	1.586214	3.147249

-4.554079	2.611426	2.337009
-5.918059	3.117365	0.507615
-6.740900	2.562143	-0.481609
-7.574762	-0.855972	-1.142227
-7.574754	0.530962	-1.325123
-6.235086	-2.780487	-1.151124
-4.554214	-3.329749	1.093133
-3.689538	-2.707730	2.078319
-2.739322	-0.314579	3.165833
-4.554050	3.499171	0.192887
-6.740965	-1.698096	-1.977956
-6.740871	1.127533	-2.350502
-6.235022	2.387284	-1.832472
-2.739283	1.124062	2.976207
-3.689422	3.153791	1.305764
-2.351596	2.707654	1.121102
-1.837893	1.713682	2.074782
-2.351634	-2.324886	1.784428
-1.837980	-1.117783	2.447984
-4.086215	-3.518880	-0.199856
-4.932685	-3.233567	-1.338693
-5.918056	-1.119102	-2.953362
-5.918020	0.315373	-3.142404
-4.932590	2.776184	-2.131008
-4.086114	3.346751	-1.104945
-4.086095	1.932458	-2.947340
-2.739277	2.015307	-2.461510
-2.739275	2.898869	-1.310464
-1.837885	2.678870	-0.256009
-0.618196	0.950314	1.901153
-0.618213	2.016032	-0.672571
-0.618321	-0.425597	2.082209
-0.618223	-2.121558	-0.127493
-1.837901	-2.653689	0.446759
-2.739359	-3.139563	-0.514550
-4.086171	-2.630345	-2.345854
-2.739302	-2.584520	-1.855249
-4.554074	-1.582606	-3.126728
-4.554044	0.718133	-3.430028
-3.689437	-0.446129	-3.384011
-2.351656	-0.382975	-2.905296
-1.837893	0.939829	-2.521393
-0.618154	1.171168	-1.773538
-0.618231	-1.590538	-1.409612
-1.838010	-1.561152	-2.191704
7.574760	-0.530951	1.325115
7.574710	-1.417317	0.170351
6.740911	-2.562135	0.481602

6.235033	-2.387275	1.832467
6.740882	-1.127523	2.350496
5.918033	-0.315365	3.142403
4.932599	-2.776173	2.131002
7.574759	0.855984	1.142219
7.574694	-0.882219	-1.122140
5.918074	-3.117362	-0.507620
6.740961	1.698104	1.977950
5.918060	1.119110	2.953357
4.554060	-0.718127	3.430031
4.086107	-1.932445	2.947333
4.086126	-3.346741	1.104940
4.554065	-3.499173	-0.192890
5.918174	-2.564010	-1.844529
6.740920	-1.471900	-2.151606
7.574728	1.412814	-0.202774
7.574729	0.561022	-1.312418
6.235072	2.780492	1.151118
4.554074	1.582614	3.126730
3.689450	0.446134	3.384013
2.739288	-2.015294	2.461503
4.554085	-2.611431	-2.337013
6.740957	2.599225	-0.198803
6.740870	0.863875	-2.459459
6.234899	-0.393330	-2.983358
2.739285	-2.898861	1.310457
3.689437	-3.153801	-1.305766
2.351611	-2.707661	-1.121099
1.837895	-2.678872	0.256005
2.351667	0.382980	2.905297
1.837898	-0.939827	2.521390
4.086162	2.630352	2.345859
4.932673	3.233572	1.338688
5.918226	2.879295	-1.298119
5.918203	1.998275	-2.445828
4.932575	-0.457343	-3.469617
4.086118	-1.586221	-3.147252
4.086185	0.716561	-3.450838
2.739313	0.314571	-3.165826
2.739281	-1.124072	-2.976206
1.837904	-1.713693	-2.074771
0.618214	-2.016030	0.672573
0.618195	-0.950341	-1.901130
0.618156	-1.171179	1.773542
0.618241	1.590500	1.409609
1.838028	1.561150	2.191731
2.739287	2.584544	1.855277
4.086198	3.518891	0.199857

2.739338	3.139575	0.514550
4.554193	3.329739	-1.093132
4.554191	1.916642	-2.933794
3.689523	2.707722	-2.078320
2.351621	2.324863	-1.784415
1.837966	1.117766	-2.447975
0.618311	0.425569	-2.082189
0.618215	2.121503	0.127520
1.837913	2.653675	-0.446751

Table S5. Cartesian coordinates of *trans*-1 $(C_{60})_3$

-10.535769	-1.176819	3.026695
-9.804691	-0.000023	3.467102
-10.535769	1.176780	3.026710
-11.708731	0.727904	2.304824
-11.708731	-0.727933	2.304815
-12.145427	-1.426975	1.174737
-11.424985	-2.604809	0.726844
-10.295314	-3.041190	1.425823
-9.840610	-2.312815	2.598978
-8.390219	-2.315395	2.592246
-7.683889	-1.175886	3.000616
-8.408375	-0.000023	3.445703
-7.683888	1.175846	3.000631
-8.390217	2.315361	2.592276
-9.840609	2.312781	2.599009
-10.295312	3.041173	1.425863
-11.424984	2.604801	0.726879
-12.145427	1.426961	1.174756
-12.591806	0.699076	0.000011
-12.591806	-0.699074	0.000002
-12.145430	-1.426959	-1.174744
-11.424987	-2.604800	-0.726869
-10.295317	-3.041171	-1.425856
-9.122793	-3.492423	-0.698218
-9.122792	-3.492433	0.698177
-7.948697	-3.040061	1.425504
-6.821087	-2.590917	0.725716
-6.069646	-1.450747	1.176392
-6.509919	-0.740190	2.276504
-6.509918	0.740158	2.276513
-6.069643	1.450731	1.176412
-6.821086	2.590907	0.725750
-7.948695	3.040042	1.425544
-9.122790	3.492424	0.698223

-9.122792	3.492434	-0.698172
-10.295315	3.041192	-1.425815
-11.424985	2.604811	-0.726834
-12.145429	1.426977	-1.174725
-11.708736	0.727935	-2.304804
-11.708736	-0.727902	-2.304814
-10.535776	-1.176779	-3.026702
-9.840616	-2.312780	-2.599002
-8.390224	-2.315360	-2.592273
-7.948700	-3.040042	-1.425542
-6.821088	-2.590907	-0.725750
-6.069646	-1.450732	-1.176413
-5.355457	-0.800898	-0.000006
-5.355457	0.800897	0.000003
-6.069648	1.450746	-1.176394
-6.821088	2.590917	-0.725716
-7.948699	3.040061	-1.425502
-8.390223	2.315395	-2.592242
-9.840615	2.312816	-2.598971
-10.535775	1.176821	-3.026687
-9.804699	0.000024	-3.467095
-8.408383	0.000023	-3.445700
-7.683895	-1.175846	-3.000629
-6.509924	-0.740159	-2.276513
-6.509923	0.740189	-2.276504
-7.683895	1.175886	-3.000614
7.683898	1.175850	-3.000629
8.408387	-0.000018	-3.445701
7.683899	-1.175882	-3.000617
6.509926	-0.740186	-2.276508
6.509926	0.740162	-2.276516
6.069647	1.450733	-1.176415
6.821088	2.590908	-0.725749
7.948700	3.040044	-1.425539
8.390226	2.315364	-2.592270
9.840618	2.312785	-2.598998
10.535779	1.176785	-3.026699
9.804703	-0.000018	-3.467095
10.535779	-1.176815	-3.026687
9.840619	-2.312811	-2.598974
8.390227	-2.315391	-2.592246
7.948702	-3.040059	-1.425507
6.821090	-2.590916	-0.725722
6.069649	-1.450746	-1.176399
5.355457	-0.800898	-0.000003
5.355457	0.800897	-0.000010

6.069643	1.450745	1.176391
6.821085	2.590916	0.725717
7.948694	3.040060	1.425507
9.122790	3.492432	0.698181
9.122793	3.492425	-0.698213
10.295317	3.041175	-1.425850
11.424987	2.604802	-0.726863
12.145431	1.426963	-1.174739
11.708738	0.727907	-2.304810
11.708739	-0.727930	-2.304802
12.145431	-1.426973	-1.174723
11.424988	-2.604808	-0.726835
10.295318	-3.041189	-1.425818
9.122794	-3.492432	-0.698176
9.122791	-3.492424	0.698218
7.948695	-3.040044	1.425539
6.821086	-2.590908	0.725744
6.069643	-1.450733	1.176406
6.509916	-0.740162	2.276509
6.509916	0.740186	2.276502
7.683885	1.175882	3.000616
8.390215	2.315392	2.592248
9.840606	2.312812	2.598982
10.295311	3.041190	1.425829
11.424983	2.604810	0.726851
12.145425	1.426975	1.174742
12.591806	0.699076	0.000007
12.591806	-0.699074	0.000014
12.145426	-1.426961	1.174757
11.424984	-2.604801	0.726878
10.295312	-3.041173	1.425860
9.840607	-2.312784	2.599006
8.390216	-2.315364	2.592272
7.683885	-1.175850	3.000628
8.408372	0.000018	3.445703
9.804687	0.000019	3.467103
10.535765	1.176816	3.026698
11.708728	0.727932	2.304819
11.708728	-0.727906	2.304826
10.535766	-1.176783	3.026711
-1.426875	1.176768	-2.995202
-0.698327	-0.000004	-3.431487
-1.426875	-1.176774	-2.995200
-2.601581	-0.740666	-2.275108
-2.601581	0.740660	-2.275109
-3.045924	1.452554	-1.176004

-2.298434	2.593916	-0.726112
-1.171664	3.047200	-1.426216
-0.724959	2.320328	-2.590775
0.724956	2.320328	-2.590776
1.426872	1.176768	-2.995204
0.698323	-0.000003	-3.431488
1.426872	-1.176774	-2.995201
0.724956	-2.320334	-2.590772
-0.724959	-2.320334	-2.590772
-1.171664	-3.047204	-1.426212
-2.298434	-2.593919	-0.726108
-3.045923	-1.452558	-1.176002
-3.759356	-0.801736	-0.000003
-3.759356	0.801735	-0.000002
-3.045921	1.452556	1.175995
-2.298433	2.593917	0.726101
-1.171662	3.047202	1.426203
0.000000	3.500436	0.698013
-0.000001	3.500435	-0.698029
1.171662	3.047200	-1.426218
2.298433	2.593916	-0.726115
3.045921	1.452554	-1.176008
2.601578	0.740661	-2.275113
2.601579	-0.740665	-2.275111
3.045923	-1.452557	-1.176005
2.298433	-2.593919	-0.726111
1.171663	-3.047203	-1.426213
0.000000	-3.500438	-0.698023
0.000000	-3.500436	0.698019
-1.171663	-3.047201	1.426208
-2.298433	-2.593918	0.726105
-3.045923	-1.452556	1.175997
-2.601578	-0.740661	2.275101
-2.601579	0.740664	2.275102
-1.426872	1.176773	2.995192
-0.724956	2.320333	2.590763
0.724959	2.320333	2.590762
1.171664	3.047202	1.426202
2.298434	2.593917	0.726098
3.045924	1.452556	1.175992
3.759355	0.801735	-0.000009
3.759356	-0.801736	-0.000006
3.045923	-1.452556	1.175994
2.298434	-2.593918	0.726102
1.171664	-3.047201	1.426206
0.724959	-2.320330	2.590765

-0.724956	-2.320330	2.590766
-1.426872	-1.176769	2.995193
-0.698323	0.000002	3.431478
0.698327	0.000002	3.431477
1.426875	1.176773	2.995190
2.601581	0.740664	2.275098
2.601581	-0.740662	2.275099
1.426875	-1.176769	2.995192

Table S6. Cartesian coordinates of *trans*-2 $(C_{60})_3$

9.920596	-0.923041	3.330701
9.062623	-2.096716	3.334017
9.425761	-3.227720	2.595444
10.655934	-3.231704	1.830485
11.480543	-2.101896	1.824803
11.105450	-0.925914	2.588509
9.068584	0.252668	3.330930
7.687778	-0.200752	3.333615
7.684899	-1.643399	3.335240
6.719154	-2.328963	2.585916
8.422023	-3.950270	1.831179
10.416631	-3.963648	0.595033
11.011496	-3.536801	-0.596706
11.862026	-2.359510	-0.601562
12.091596	-1.656463	0.584854
12.092239	-0.204158	0.581705
11.483922	0.248223	1.819478
10.663037	1.380174	1.822298
9.432123	1.380597	2.592350
6.723369	0.485808	2.584467
7.101048	1.658307	1.816775
8.429926	2.101910	1.825647
9.034840	2.543912	0.592683
10.416050	2.101244	0.584312
11.001983	1.661438	-0.607193
11.856272	0.491515	-0.608498
11.617405	-0.240433	-1.844012
11.623810	-1.639254	-1.840041
9.038876	-4.411285	0.598076
8.284146	-2.132256	-3.335042
7.920476	-3.260112	-2.596443
6.688305	-3.258394	-1.825598
5.871509	-2.120207	-1.812523
6.249837	-0.947944	-2.580162
8.273834	0.214979	-3.336730

9.655847	-0.224952	-3.340641
9.663388	-1.678670	-3.338957
10.627943	-2.371508	-2.600999
10.249408	-3.545680	-1.831989
8.920894	-3.981784	-1.830586
6.923762	-3.972167	-0.594130
6.332664	-3.511575	0.590149
5.475727	-2.346106	0.579037
5.226500	-1.679338	-0.605093
4.960794	-0.182246	-0.675015
5.840238	0.220784	-1.849484
6.661732	1.331407	-1.829929
7.895569	1.335650	-2.584955
10.615299	0.477908	-2.604540
10.228015	1.650809	-1.837748
8.894027	2.063792	-1.824084
8.282007	2.518456	-0.589179
6.904856	2.075824	-0.573696
6.316219	1.680016	0.611906
5.223771	0.622549	0.684336
5.703908	-0.220451	1.856603
5.718876	-1.601863	1.835338
8.302372	-4.422889	-0.591030
7.098997	-3.503250	1.822488
7.430758	-0.955792	-3.333594
0.387437	0.706928	3.341221
-0.994480	1.151673	3.335718
-1.951132	0.461148	2.579411
-1.561491	-0.707473	1.810833
-0.226732	-1.132634	1.816581
0.767799	-0.410244	2.587983
1.228248	1.877651	3.344886
0.369890	3.052438	3.342561
-1.004824	2.595527	3.336785
-1.968239	3.275842	2.577600
-2.968436	1.160073	1.846339
-2.347797	-0.742338	0.607158
-1.760743	-1.145105	-0.576018
-0.380492	-1.580649	-0.589633
0.380451	-1.580641	0.589709
1.760717	-1.145142	0.576086
2.004282	-0.405901	1.836575
2.824846	0.705952	1.860691
2.411146	1.872966	2.593199
0.735437	4.180341	2.602002
1.967662	4.183437	1.840563

2.787448	3.045668	1.827672
3.437273	2.610113	0.622046
3.704576	1.113848	0.688230
3.442241	0.311788	-0.674531
2.347743	-0.742373	-0.607108
1.561457	-0.707528	-1.810781
0.226694	-1.132675	-1.816514
-3.442258	0.311914	0.674555
-1.228236	1.877596	-3.344881
-2.411138	1.872949	-2.593187
-2.787432	3.045676	-1.827692
-1.967638	4.183435	-1.840621
-0.735399	4.180285	-2.602043
1.004844	2.595442	-3.336803
0.994476	1.151580	-3.335709
-0.387450	0.706869	-3.341190
-0.767833	-0.410298	-2.587940
-2.004296	-0.405914	-1.836530
-2.824838	0.705966	-1.860655
-3.437297	2.610158	-0.622061
-3.195148	3.281812	0.561700
-2.340537	4.449056	0.574085
-1.739391	4.903550	-0.608057
-0.364145	5.353676	-0.597449
0.262939	4.899903	-1.829144
1.585062	4.448690	-1.811943
1.968267	3.275749	-2.577626
1.951121	0.461066	-2.579397
2.968470	1.159978	-1.846346
2.958582	2.543682	-1.821459
3.195205	3.281762	-0.561739
2.340581	4.449002	-0.574141
1.739434	4.903532	0.607988
0.364202	5.353687	0.597376
-0.262893	4.899950	1.829081
-1.585022	4.448766	1.811893
-3.704567	1.113840	-0.688209
-2.958567	2.543783	1.821450
-0.369865	3.052376	-3.342591
-8.429880	2.101630	-1.826085
-9.034859	2.543807	-0.593220
-8.282089	2.518550	0.588688
-6.904928	2.075949	0.573347
-6.316240	1.679945	-0.612172
-7.100992	1.658052	-1.817077
-9.432027	1.380207	-2.592747

-10.662981	1.379877	-1.822757
-10.416066	2.101130	-0.584861
-11.002057	1.661500	0.606680
-8.894171	2.064050	1.823623
-6.661860	1.331693	1.829685
-5.840326	0.221112	1.849482
-4.960828	-0.182133	0.675111
-5.223755	0.622554	-0.684381
-5.703789	-0.220675	-1.856558
-6.723250	0.485449	-2.584579
-7.687604	-0.201228	-3.333677
-9.068419	0.252165	-3.331120
-11.483849	0.247917	-1.819811
-11.105309	-0.926330	-2.588644
-9.920414	-0.923555	-3.330770
-9.062425	-2.097220	-3.333870
-7.684708	-1.643885	-3.335084
-6.719000	-2.329311	-2.585598
-5.718774	-1.602076	-1.835062
-5.475668	-2.346155	-0.578653
-5.226550	-1.679194	0.605399
-7.895742	1.336041	2.584659
-7.920592	-3.259722	2.596832
-8.284316	-2.131756	3.335239
-9.663566	-1.678187	3.339014
-10.628067	-2.371153	2.601107
-10.249480	-3.545435	1.832292
-8.302363	-4.422798	0.591570
-6.923756	-3.972055	0.594678
-6.688389	-3.258091	1.826046
-5.871602	-2.119894	1.812860
-6.249983	-0.947524	2.580304
-7.430939	-0.955285	3.333670
-9.656043	-0.224468	3.340483
-10.615462	0.478268	2.604215
-11.617519	-0.240198	1.843744
-11.623898	-1.639020	1.839982
-11.862041	-2.359462	0.601599
-11.011500	-3.536748	0.596966
-10.416563	-3.963759	-0.594678
-9.038802	-4.411379	-0.597577
-6.332599	-3.511624	-0.589636
-7.098866	-3.503490	-1.822017
-8.421886	-3.950534	-1.830714
-9.425589	-3.228112	-2.595140
-10.655806	-3.232007	-1.830252

-11.480424	-2.102204	-1.824778
-12.091561	-1.656593	-0.584933
-12.092224	-0.204290	-0.582004
-11.856333	0.491563	0.608110
-8.274040	0.215477	3.336571
-10.228158	1.651063	1.837281
-8.920961	-3.981521	1.831029

Table S7. Cartesian coordinates of *trans*-3 (C_{60})$_3$

-10.096597	-0.073006	-2.409966
-9.157412	-0.800487	-3.248111
-9.158141	-2.199256	-3.243864
-10.094742	-2.922879	-2.408460
-10.996806	-2.223583	-1.600012
-10.997073	-0.772136	-1.600420
-9.416114	1.099973	-1.891206
-8.058970	1.091202	-2.411179
-7.900692	-0.076279	-3.243942
-6.686718	-0.776241	-3.221370
-7.899621	-2.927141	-3.238026
-9.418697	-4.103719	-1.891018
-9.671928	-4.538493	-0.586003
-10.604048	-3.807024	0.253896
-11.253269	-2.672874	-0.242923
-11.410195	-1.497868	0.596267
-11.252817	-0.323101	-0.241679
-10.597640	0.807611	0.255537
-9.661465	1.531183	-0.585971
-6.994744	1.502738	-1.598272
-7.249746	1.951299	-0.241608
-8.560043	1.971856	0.253702
-8.811979	1.525110	1.602227
-10.070886	0.804879	1.610554
-10.222259	-0.329447	2.414897
-10.905147	-1.498998	1.900652
-10.229156	-2.680022	2.417983
-10.084955	-3.812853	1.610315
-8.065218	-4.108672	-2.407264
-6.418421	-4.135259	1.896388
-6.172996	-4.566586	0.591228
-5.235980	-3.841269	-0.250361
-4.588454	-2.701138	0.243144
-4.843927	-2.252701	1.599729
-6.661820	-2.231661	3.247981
-7.923327	-2.947526	3.254458

-7.773727	-4.126513	2.417101
-8.834093	-4.549136	1.609552
-8.578593	-4.998270	0.250711
-7.272500	-5.007444	-0.248350
-5.750663	-3.833032	-1.598130
-5.594663	-2.684213	-2.385581
-4.905157	-1.524167	-1.864537
-4.386603	-1.542179	-0.584047
-4.275507	-0.297154	0.284236
-4.800565	-0.815394	1.615084
-5.705517	-0.117656	2.391581
-6.649578	-0.830440	3.224323
-9.129351	-2.238933	3.251354
-9.118674	-0.785191	3.244420
-7.902775	-0.098610	3.223208
-7.741618	1.077456	2.388437
-6.392912	1.083139	1.865479
-6.147311	1.538148	0.584331
-5.019299	1.001873	-0.285104
-5.734102	0.811110	-1.614709
-5.592649	-0.323314	-2.390494
-7.009690	-4.553036	-1.604138
-6.691227	-2.227400	-3.219219
-5.736966	-2.960743	2.414444
-1.242978	3.732053	-3.243895
0.018747	4.449107	-3.239696
1.223053	3.740300	-3.214302
1.217198	2.287680	-3.209848
-0.002968	1.600150	-3.215016
-1.254443	2.329277	-3.224468
-2.169168	4.458072	-2.416108
-1.492509	5.638744	-1.896872
-0.139062	5.634863	-2.410765
0.917863	6.070578	-1.603940
2.318582	4.189481	-2.382703
2.313893	1.828046	-2.384784
2.173457	0.689540	-1.613486
0.911290	0.004205	-1.592442
-0.164462	0.431641	-2.386940
-1.515701	0.416191	-1.867184
-2.201754	1.615127	-2.394633
-3.107478	2.313313	-1.617752
-3.064878	3.749783	-1.602564
-1.746530	6.073571	-0.593372
-2.680829	5.343327	0.246474
-3.322295	4.199000	-0.247489

-3.523019	3.040509	0.580039
-3.633343	1.795189	-0.287019
-2.889392	0.496116	0.283433
-1.761159	-0.038273	-0.587070
-0.658546	-0.440670	0.243676
0.658597	-0.440631	-0.243913
3.003339	3.023885	-1.860306
2.201773	1.615006	2.394513
3.107524	2.313239	1.617718
3.064871	3.749722	1.602626
2.169158	4.457936	2.416215
1.242970	3.731855	3.243951
0.002994	1.599928	3.214933
0.164506	0.431488	2.386775
1.515743	0.416074	1.867038
1.761169	-0.038232	0.586847
2.889426	0.496171	-0.283543
3.633370	1.795240	0.286879
3.322280	4.199033	0.247578
2.680778	5.343379	-0.246317
1.746488	6.073566	0.593585
1.492465	5.638628	1.897041
0.139020	5.634701	2.410950
-0.018768	4.448885	3.239788
-1.223062	3.740058	3.214360
-1.217187	2.287442	3.209816
-0.911232	0.004081	1.592226
-2.173375	0.689429	1.613300
-2.313885	1.827839	2.384745
-3.003320	3.023693	1.860286
-2.318588	4.189276	2.382768
-2.170203	5.340688	1.594769
-0.917903	6.070448	1.604146
-0.654207	6.525336	0.247724
0.654146	6.525355	-0.247485
3.522982	3.040612	-0.580028
2.170165	5.340832	-1.594603
1.254456	2.329081	3.224421
8.560065	1.971821	-0.254270
8.811915	1.524852	-1.602747
7.741511	1.077080	-2.388821
6.392823	1.082858	-1.865769
6.147284	1.538126	-0.584716
7.249805	1.951365	0.241100
9.661539	1.531246	0.585400
10.597659	0.807540	-0.256062

10.070802	0.804600	-1.611037
10.222121	-0.329856	-2.415210
7.902595	-0.099102	-3.223434
5.705374	-0.117978	-2.391704
4.800531	-0.815622	-1.614978
4.275514	-0.297140	-0.284278
5.019341	1.001952	0.284937
5.734274	0.811367	1.614456
6.994886	1.502997	1.597852
8.059153	1.091558	2.410763
9.416271	1.100246	1.890716
11.252829	-0.323119	0.241291
10.997184	-0.771938	1.600111
10.096764	-0.072661	2.409607
9.157627	-0.800004	3.247921
7.900914	-0.075771	3.243733
6.686932	-0.775728	3.221312
5.592792	-0.322908	2.390465
4.905279	-1.523833	1.864702
4.386596	-1.542039	0.584274
6.649386	-0.830918	-3.224372
6.172917	-4.566636	-0.590652
6.418257	-4.135551	-1.895901
7.773537	-4.126901	-2.416693
8.833946	-4.549390	-1.609132
8.578519	-4.998306	-0.250206
7.009744	-4.552771	1.604665
5.750729	-3.832758	1.598631
5.235935	-3.841203	0.250897
4.588427	-2.701130	-0.242757
4.843830	-2.252912	-1.599417
5.736813	-2.961086	-2.414093
7.923106	-2.948026	-3.254231
9.129143	-2.239451	-3.251319
10.228995	-2.680435	-2.417953
10.084812	-3.813135	-1.610095
10.604009	-3.807105	-0.253714
9.671931	-4.538432	0.586368
9.418781	-4.103442	1.891321
8.065328	-4.108292	2.407654
5.594794	-2.683818	2.385909
6.691418	-2.226876	3.219399
7.899809	-2.926636	3.238236
9.158345	-2.198773	3.243899
10.094879	-2.922544	2.408548
10.996901	-2.223382	1.599936

11.253271	-2.672895	0.242894
11.410158	-1.498009	-0.596483
10.905028	-1.499340	-1.900837
6.661609	-2.232146	-3.247794
9.118481	-0.785714	-3.244589
7.272461	-5.007395	0.248928

Table S8. Cartesian coordinates of *trans*-4 $(C_{60})_3$

-6.670910	-4.758382	1.830376
-5.954257	-3.759014	2.602248
-6.659826	-2.800527	3.332522
-8.111613	-2.800961	3.321643
-8.803617	-3.761558	2.577724
-8.069212	-4.759527	1.817485
-5.933026	-4.985147	0.598441
-4.764638	-4.125965	0.612421
-4.776543	-3.373100	1.842991
-4.356344	-2.036577	1.832742
-6.217127	-1.416250	3.334049
-8.559027	-1.417759	3.317259
-9.682704	-1.051109	2.569237
-10.401831	-2.047673	1.802319
-9.970568	-3.378277	1.804310
-9.959140	-4.138357	0.566832
-8.783285	-4.990799	0.575130
-8.072629	-5.211254	-0.609191
-6.624150	-5.205661	-0.597464
-4.334922	-3.505180	-0.568301
-5.053028	-3.738087	-1.807666
-6.169828	-4.576385	-1.826659
-7.341300	-4.204756	-2.603235
-8.516266	-4.592261	-1.849840
-9.652982	-3.777185	-1.856986
-10.385296	-3.543845	-0.624681
-10.840084	-2.164517	-0.627804
-10.845400	-1.428507	0.561632
-7.383904	-0.567725	3.324338
-8.794771	1.401461	-0.622040
-8.083776	1.613916	0.566805
-6.637357	1.624203	0.566133
-5.942394	1.431324	-0.612321
-6.668757	1.163997	-1.824332
-8.788122	0.170170	-2.620247
-9.965004	-0.214226	-1.861719
-9.968364	0.549281	-0.624595

-10.399744	-0.050162	0.563322
-9.673732	0.183105	1.801056
-8.535423	0.992164	1.797768
-6.186270	0.994748	1.827669
-5.058629	0.196996	1.859870
-4.085546	0.087486	0.695260
-4.573705	0.768381	-0.669702
-4.753977	-0.183741	-1.842851
-5.935543	0.167415	-2.583358
-6.629342	-0.787185	-3.338556
-8.082606	-0.788302	-3.350585
-10.390993	-1.546040	-1.861820
-9.656519	-2.543807	-2.622207
-8.524208	-2.171417	-3.353192
-7.342397	-3.018057	-3.343513
-6.175619	-2.156605	-3.333783
-5.051540	-2.504912	-2.572631
-4.336812	-1.500655	-1.815668
-3.885540	-2.130298	-0.554146
-3.869609	-1.417635	0.629444
-7.367218	0.613833	2.571064
-5.089885	-1.040310	2.591841
-8.069691	1.169831	-1.847555
0.702891	0.635310	1.844243
1.428231	1.630900	2.613532
0.727990	2.582591	3.362193
-0.727950	2.582606	3.362213
-1.428229	1.630943	2.613561
-0.702928	0.635334	1.844259
1.427313	0.365533	0.646220
2.796005	1.028070	0.704822
2.612797	1.982255	1.877583
3.024024	3.301199	1.847796
1.174119	3.952817	3.356089
-1.174044	3.952849	3.356120
-2.301123	4.304971	2.598704
-3.023983	3.301273	1.847846
-2.612812	1.982315	1.877640
-2.796061	1.028148	0.704877
-1.427382	0.365568	0.646250
-0.721964	0.183107	-0.532309
0.721867	0.183076	-0.532324
3.283513	1.709566	-0.660388
2.310515	1.598754	-1.824648
1.176253	0.811243	-1.788366
-0.000059	1.188839	-2.539977

-1.176354	0.811282	-1.788334
-2.310607	1.598814	-1.824598
-3.283580	1.709662	-0.660299
-3.497833	3.214147	-0.596044
-3.475432	3.928563	0.584858
0.000051	4.806473	3.353006
-1.425500	6.778515	-0.585980
-0.723072	6.994636	0.604898
0.723171	6.994618	0.604883
1.425564	6.778478	-0.586010
0.699572	6.552580	-1.823979
-1.424390	5.553171	-2.589833
-2.594241	5.167809	-1.820649
-2.595356	5.921241	-0.589861
-3.018507	5.302608	0.593850
-2.294747	5.533662	1.828822
-1.171529	6.366761	1.839223
1.171636	6.366730	1.839199
2.294833	5.533605	1.828775
3.018551	5.302523	0.593784
2.595393	5.921166	-0.589916
2.594244	5.167745	-1.820707
1.424374	5.553129	-2.589853
0.725423	4.593473	-3.326256
-0.725469	4.593490	-3.326230
-3.015512	3.831005	-1.805135
-2.287106	2.834643	-2.562782
-1.167548	3.208331	-3.318312
-0.000053	2.363009	-3.309081
1.167464	3.208305	-3.318331
2.287034	2.834585	-2.562838
3.015468	3.830926	-1.805194
3.497823	3.214055	-0.596125
3.475425	3.928457	0.584784
0.000056	5.989687	2.605050
2.301205	4.304916	2.598670
-0.699539	6.552593	-1.823961
8.524190	-2.171513	-3.353189
8.082554	-0.788407	-3.350626
8.788052	0.170109	-2.620329
9.964953	-0.214226	-1.861799
10.390971	-1.546031	-1.861856
9.656518	-2.543849	-2.622202
7.342402	-3.018179	-3.343471
6.175601	-2.156758	-3.333761
6.629292	-0.787324	-3.338577

5.935473	0.167289	-2.583417
8.069607	1.169785	-1.847667
9.968308	0.549330	-0.624704
10.399705	-0.050063	0.563231
10.845394	-1.428396	0.561590
10.840089	-2.164448	-0.627819
10.385332	-3.543786	-0.624642
9.653022	-3.777195	-1.856933
8.516323	-4.592298	-1.849747
7.341340	-4.204851	-2.603146
5.051540	-2.505067	-2.572581
5.053066	-3.738207	-1.807561
6.169884	-4.576486	-1.826550
6.624236	-5.205693	-0.597335
8.072716	-5.211263	-0.609077
8.783360	-4.990733	0.575239
9.959197	-4.138272	0.566892
9.970625	-3.378141	1.804342
10.401852	-2.047527	1.802306
8.794690	1.401481	-0.622171
7.383904	-0.567603	3.324292
7.367180	0.613930	2.570976
6.186219	0.994784	1.827567
5.058590	0.197012	1.859821
5.089892	-1.040265	2.591829
6.659885	-2.800419	3.332561
8.111671	-2.800819	3.321679
8.559051	-1.417606	3.317240
9.682708	-1.050952	2.569191
9.673703	0.183237	1.800964
8.535369	0.992260	1.797656
6.637278	1.624207	0.566019
5.942314	1.431266	-0.612428
4.573639	0.768283	-0.669774
4.085489	0.087432	0.695216
3.869604	-1.417701	0.629461
4.356370	-2.036584	1.832773
4.776602	-3.373091	1.843076
5.954327	-3.758950	2.602335
8.803694	-3.761422	2.577786
8.069307	-4.759446	1.817606
6.671002	-4.758326	1.830476
5.933110	-4.985158	0.598575
4.764714	-4.126007	0.612525
4.334956	-3.505269	-0.568210
3.885543	-2.130395	-0.554105

4.336782	-1.500797	-1.815671
4.753932	-0.183877	-1.842877
8.083700	1.613958	0.566672
6.668670	1.163916	-1.824432
6.217151	-1.416153	3.334042

Table S9. Cartesian coordinates of *e*-(C_{60})$_3$

-0.529688	3.813507	-3.456397
-1.872474	4.128952	-3.008950
-2.179013	5.433480	-2.596695
-1.151527	6.458241	-2.603226
0.142852	6.147890	-3.035155
0.456107	4.800431	-3.478404
-0.210801	2.470966	-3.005627
-1.337260	1.951033	-2.280334
-2.388387	2.987742	-2.282175
-3.198281	3.177914	-1.176987
-2.998732	5.629217	-1.426913
-1.341979	7.290195	-1.427563
-0.232433	7.777366	-0.728305
1.105976	7.453953	-1.175742
1.292104	6.655030	-2.310758
2.316113	5.624915	-2.313504
1.805742	4.487322	-3.034149
2.116140	3.196163	-2.588823
1.091815	2.168723	-2.577428
-1.125957	1.144421	-1.173377
0.210897	0.866113	-0.736834
1.310317	1.322590	-1.448377
2.646901	1.649295	-0.799981
2.971058	2.986444	-1.450836
3.433668	4.077165	-0.740526
3.108018	5.418297	-1.175093
2.907600	6.243989	-0.000021
1.933439	7.247137	-0.000020
-2.487483	6.777055	-0.698628
0.142879	6.147915	3.035143
-1.151502	6.458262	2.603223
-2.178992	5.433501	2.596709
-1.872451	4.128973	3.008978
-0.529658	3.813529	3.456404
1.805763	4.487342	3.034122
2.316129	5.624930	2.313470
1.292125	6.655050	2.310730
1.105988	7.453964	1.175709

-0.232426	7.777374	0.728284
-1.341964	7.290212	1.427557
-2.998707	5.629231	1.426934
-3.474859	4.511329	0.726903
-3.198229	3.177956	1.177011
-2.388379	2.987760	2.282235
-1.337246	1.951057	2.280331
-0.210779	2.470988	3.005627
1.091833	2.168744	2.577427
2.116157	3.196179	2.588806
3.108024	5.418301	1.175053
3.433667	4.077166	0.740491
2.971067	2.986448	1.450812
2.646891	1.649298	0.799970
1.310319	1.322594	1.448378
0.210892	0.866100	0.736869
-1.125983	1.144379	1.173421
-2.088615	1.093509	-0.000019
-3.233536	2.214056	0.000061
-2.487480	6.777072	0.698638
-3.474872	4.511317	-0.726884
0.456138	4.800452	3.478392
5.948119	-2.635237	-3.492962
4.603574	-2.319496	-3.040193
4.300989	-1.027434	-2.590926
5.327047	-0.001007	-2.590763
6.619165	-0.303113	-3.040086
6.935373	-1.647582	-3.492829
6.263018	-3.978810	-3.041315
5.111987	-4.486954	-2.312926
4.090925	-3.456889	-2.315609
3.302559	-3.246542	-1.176073
3.451220	-0.815137	-1.450374
5.114308	0.848809	-1.450173
6.203493	1.315931	-0.740017
7.545733	0.998131	-1.175939
7.756396	0.209908	-2.315464
8.786782	-0.810824	-2.312795
8.279064	-1.962011	-3.041222
8.583701	-3.254932	-2.604605
7.556018	-4.282988	-2.604639
5.301017	-5.280949	-1.176956
6.640921	-5.599563	-0.727910
7.748761	-5.109178	-1.426819
8.895075	-4.593943	-0.698982
9.409953	-3.447431	-1.426799

9.899904	-2.339435	-0.727854
9.580887	-0.999609	-1.176854
9.375465	-0.171175	0.000050
8.372907	0.800943	0.000049
3.778140	0.521247	-0.800365
6.619103	-0.303170	3.040127
5.326994	-0.001057	2.590782
4.300935	-1.027483	2.590904
4.603511	-2.319552	3.040152
5.948046	-2.635302	3.492944
8.279001	-1.962068	3.041267
8.786734	-0.810867	2.312871
7.756348	0.209864	2.315537
7.545709	0.998109	1.176022
6.203478	1.315916	0.740078
5.114279	0.848780	1.450202
3.451188	-0.815160	1.450337
2.984350	-1.904416	0.740169
3.302536	-3.246562	1.175987
4.090878	-3.456931	2.315536
5.111939	-4.486997	2.312857
6.262954	-3.978866	3.041280
7.555964	-4.283037	2.604626
8.583647	-3.254981	2.604633
9.580863	-0.999631	1.176944
9.899889	-2.339449	0.727926
9.409923	-3.447458	1.426840
8.895060	-4.593957	0.698992
7.748731	-5.109205	1.426794
6.640906	-5.599577	0.727853
5.300993	-5.280971	1.176877
4.472495	-5.075808	-0.000047
3.500023	-4.073624	-0.000048
3.778130	0.521231	0.800360
2.984363	-1.904404	-0.740237
6.935300	-1.647647	3.492852
-6.894017	-2.663629	-3.467904
-8.246107	-2.361844	-3.026787
-8.570584	-1.070353	-2.598557
-7.557206	-0.032822	-2.591851
-6.249105	-0.325994	-3.000642
-5.916492	-1.666434	-3.446409
-6.565251	-4.009528	-3.027264
-7.706847	-4.532891	-2.305191
-8.746459	-3.513837	-2.304906
-9.551146	-3.336611	-1.174636

-9.408958	-0.885412	-1.425248
-7.765408	0.789639	-1.424875
-6.655327	1.280381	-0.725235
-5.315149	1.018615	-1.176171
-5.116014	0.207174	-2.276529
-4.058886	-0.828985	-2.276810
-4.569589	-1.972376	-3.001076
-4.250211	-3.274472	-2.592821
-5.267429	-4.308225	-2.599367
-7.513319	-5.334143	-1.175161
-6.167895	-5.644169	-0.727342
-5.065536	-5.143033	-1.426310
-3.922270	-4.621772	-0.698799
-3.423414	-3.466588	-1.426050
-2.954864	-2.347126	-0.726181
-3.243815	-1.012222	-1.176423
-3.208048	-0.048090	-0.000085
-4.351203	1.073302	0.000115
-8.910294	0.267768	-0.697513
-6.248341	-0.326786	3.001062
-7.556541	-0.033503	2.592710
-8.569917	-1.071034	2.599403
-8.245324	-2.362640	3.027195
-6.893134	-2.664539	3.467903
-4.568814	-1.973163	3.000673
-4.058289	-0.829585	2.276600
-5.115415	0.206571	2.276792
-5.314815	1.018336	1.176729
-6.655133	1.280195	0.726210
-7.765037	0.789275	1.425989
-9.408582	-0.885787	1.426348
-9.887558	-1.997930	0.727315
-9.550844	-3.336920	1.175132
-8.745865	-3.514443	2.305146
-7.706260	-4.533505	2.304904
-6.564478	-4.010335	3.026829
-5.266772	-4.308911	2.598511
-4.249556	-3.275154	2.591976
-3.243563	-1.012566	1.175905
-2.954688	-2.347322	0.725244
-3.423066	-3.466967	1.424952
-3.922082	-4.621975	0.697528
-5.065175	-5.143416	1.425189
-6.167711	-5.644366	0.726369
-7.513021	-5.334454	1.174611
-8.345470	-5.143713	-0.000144

-9.343943	-4.165159	0.000113
-8.910098	0.267575	0.698784
-9.887746	-1.997740	-0.726381
-5.915618	-1.667337	3.446404

Table S10. Cartesian coordinates of *cyclic* (C_{60})$_3$

2.015878	0.168146	-0.397038
1.978130	-0.755519	-1.619769
2.992389	-0.824050	-2.585072
4.281523	-0.121492	-2.414544
4.510052	0.602657	-1.267283
3.404785	0.942339	-0.301762
1.582091	-0.783247	0.679057
0.742656	-1.896339	0.125052
1.304787	-1.961327	-1.272399
1.557658	-3.166101	-1.904249
3.281871	-2.070952	-3.243006
5.329264	-0.962463	-2.950711
6.575878	-1.039253	-2.312412
6.800030	-0.290554	-1.097293
5.764940	0.498504	-0.579194
5.500068	0.516819	0.849157
4.082885	0.652211	1.048551
3.488819	-0.112695	2.049910
2.207698	-0.827027	1.870067
0.720769	-3.257710	0.961413
1.618942	-3.234704	2.206470
2.243437	-2.074888	2.652802
3.534785	-2.118609	3.294474
4.287248	-0.923941	2.935952
5.676768	-0.986598	2.786395
6.296215	-0.260403	1.706770
7.363917	-1.080788	1.169437
7.611974	-1.098640	-0.205477
4.713046	-2.165902	-3.470929
7.321156	-4.749711	-0.685094
6.694966	-4.713223	-1.936777
5.437496	-5.408249	-2.137311
4.850713	-6.119334	-1.083178
5.498367	-6.151487	0.216773
6.930931	-4.721807	1.630793
7.681948	-3.529347	1.287951
7.925854	-3.546884	-0.144289
7.891610	-2.354826	-0.876128
7.247791	-2.316888	-2.179089

6.654532	-3.474651	-2.694578
4.615409	-4.594796	-3.012318
3.240607	-4.510750	-2.795667
2.629033	-5.244477	-1.701526
3.419480	-6.042797	-0.865459
3.178882	-6.026686	0.557964
4.461878	-6.093952	1.232541
4.668056	-5.360951	2.403088
5.929035	-4.662402	2.607014
7.404712	-2.318898	1.933597
6.362849	-2.257824	2.937504
5.639274	-3.408971	3.270086
4.202074	-3.335401	3.471064
3.604302	-4.538013	2.946990
2.355802	-4.467940	2.302118
2.150196	-5.226283	1.078965
1.311325	-4.449783	0.212544
1.576974	-4.429597	-1.142635
5.367033	-3.396214	-3.357040
2.564569	-3.232711	-2.914029
6.709114	-5.478268	0.410736
1.281639	1.585773	-0.183098
0.771674	1.960855	1.211684
1.492446	2.760775	2.103547
2.719137	3.477777	1.703038
3.152927	3.389795	0.403966
2.633443	2.339961	-0.536647
0.053286	1.823784	-0.994277
-1.186682	1.658109	-0.182730
-0.655184	2.002480	1.211998
-1.327560	2.843255	2.104101
0.807133	3.605015	3.039021
2.746261	4.751628	2.376969
3.219918	5.892449	1.710514
3.663181	5.785398	0.339721
3.612712	4.540653	-0.302844
3.167278	4.419571	-1.680653
2.482401	3.158778	-1.841066
1.301482	3.170611	-2.591696
0.071928	2.466503	-2.173074
-2.492079	2.490045	-0.535770
-2.293682	3.298686	-1.840086
-1.114475	3.241346	-2.591281
-0.601829	4.404579	-3.275500
0.857492	4.361863	-3.275729
1.589909	5.550913	-3.189947

2.766223	5.584117	-2.358595
2.805225	6.874270	-1.693126
3.246335	6.977965	-0.371222
1.564941	4.834361	3.210602
0.253533	8.655179	1.000078
0.234649	8.000928	2.237961
-0.962385	7.303768	2.672662
-2.100809	7.290280	1.858007
-2.078055	7.965227	0.570292
-0.476467	8.512531	-1.227913
0.973233	8.470162	-1.228193
1.425476	8.561813	0.149448
2.540647	7.830077	0.569414
2.524408	7.154960	1.857136
1.389012	7.234968	2.672228
-0.548690	6.097765	3.364705
-1.278265	4.917547	3.211115
-2.462787	4.904036	2.378020
-2.869054	6.070615	1.711639
-3.318237	5.989694	0.340966
-2.832616	7.155842	-0.370108
-2.398783	7.026565	-1.692169
-1.199560	7.723944	-2.131120
1.648667	7.640603	-2.131629
0.899018	6.821886	-3.060679
-0.499360	6.862784	-3.060376
-1.263415	5.634407	-3.189358
-2.435442	5.736337	-2.357568
-2.903687	4.597263	-1.679514
-3.340903	4.744151	-0.301593
-2.949006	3.568370	0.405099
-2.510371	3.630779	1.704036
0.905722	6.055221	3.364455
-0.593722	3.645975	3.039319
-0.922151	8.630489	0.149902
-0.852292	-1.849571	0.125121
-1.417265	-1.881361	-1.272398
-1.740124	-3.069230	-1.904378
-1.833340	-4.329541	-1.142906
-1.569415	-4.365333	0.212280
-0.910181	-3.210005	0.961410
-1.625084	-0.689322	0.679330
-2.002555	0.285926	-0.396649
-2.018964	-0.638175	-1.619519
-3.035502	-0.647171	-2.584821
-2.749259	-3.076689	-2.914131

-2.931331	-5.081396	-1.701883
-3.767217	-5.832113	-0.865930
-3.526136	-5.830207	0.557465
-2.452356	-5.091530	1.078563
-2.613294	-4.322641	2.301769
-1.805526	-3.134642	2.206360
-2.361049	-1.940382	2.652935
-2.252357	-0.696722	1.870273
-3.343857	1.139944	-0.301199
-4.037775	0.889777	1.049017
-3.489520	0.091218	2.050214
-4.334087	-0.671957	2.936163
-3.652780	-1.908609	3.294651
-4.390130	-3.084313	3.471005
-3.863700	-4.319736	2.946734
-4.973794	-5.078975	2.402762
-4.810824	-5.822557	1.232081
-3.498871	-4.313015	-2.795963
-7.586412	-4.313090	-0.685453
-6.959184	-4.313115	-1.937156
-6.846426	-3.078972	-2.694820
-7.370973	-1.888594	-2.179085
-8.015959	-1.888996	-0.876111
-7.875277	-3.074011	1.287783
-7.195416	-4.308474	1.630507
-7.018205	-5.076433	0.410306
-5.848896	-5.819251	0.216280
-5.200439	-5.824738	-1.083657
-5.744548	-5.080474	-2.137768
-5.556495	-3.075889	-3.357336
-4.831667	-1.885900	-3.470924
-5.376490	-0.648581	-2.950465
-6.625406	-0.652485	-2.312094
-6.805442	0.107929	-1.096949
-7.663476	-0.651294	-0.205303
-7.414696	-0.648179	1.169620
-7.527772	-1.881920	1.933589
-6.191763	-4.307892	2.606747
-5.829136	-3.073608	3.269893
-6.484099	-1.882043	2.937462
-5.724896	-0.653099	2.786601
-6.300776	0.108265	1.707071
-5.460458	0.837624	0.849591
-5.725899	0.834908	-0.578740
-4.467058	0.865636	-1.266809
-4.281329	0.129573	-2.414215

-4.876232 -4.316411 -3.012646
-3.397413 -1.874913 -3.242899
-8.119726 -3.077085 -0.144440

Table S11. Atomic contributions to the mean polarizability of $(C_{60})_3$ isomers

№	Distributed polarizability of atoms (a.u.)					
	trans-1	*trans*-2	*trans*-3	*trans*-4	*e*	*cyclic*
1	21.1728	16.1401	18.3407	9.6191	7.3314	0.0485
2	18.6633	14.0363	16.2221	6.8774	8.2509	-1.0437
3	21.1698	18.2115	18.5086	10.1747	13.4116	3.3931
4	25.4288	22.5474	22.1176	14.1515	16.3050	3.4507
5	25.4312	27.8407	27.3125	20.7483	14.7228	1.7942
6	28.8445	25.9142	26.3094	19.3135	12.4355	0.8234
7	26.0529	12.9082	15.5641	7.8156	5.0978	-1.5960
8	16.2858	8.1012	10.9382	3.9601	3.9881	0.0240
9	15.1607	9.2853	11.6448	3.0094	3.8930	-1.0186
10	10.1485	1.6787	3.5282	-3.0036	5.0157	2.1183
11	3.2797	15.1056	15.1896	7.1486	14.0186	6.4986
12	4.9056	21.9728	20.6838	14.9136	17.9454	9.0608
13	3.2785	26.1404	23.0247	21.9275	20.1879	14.3882
14	10.1513	28.6581	25.4791	24.7911	19.3197	13.7748
15	15.1674	29.8512	28.5677	24.1163	16.4192	5.4526
16	16.3032	29.2139	28.1617	24.6489	13.2996	5.3153
17	26.0661	26.7538	26.8206	21.8040	11.1756	1.2171
18	28.8472	18.7243	19.9197	17.5064	4.9463	0.5884
19	30.5146	14.7700	16.7213	13.0965	3.7939	2.2413
20	30.5144	-2.3624	0.1366	-0.5082	1.3271	0.8817
21	28.8445	-1.9700	0.3793	1.0629	0.5962	2.1395
22	26.0545	10.3511	12.3338	11.3180	2.4433	1.3334
23	16.2848	13.0722	14.0044	14.4410	0.3907	5.6347
24	12.3535	18.0699	18.6850	18.4197	3.5673	5.1903
25	12.3539	23.3631	21.6823	24.4865	7.3355	11.7439
26	7.6949	26.8659	24.7557	26.5612	12.0078	12.2820
27	-3.6662	26.3333	23.3746	26.7225	13.6761	15.8719
28	-9.3855	27.5814	23.8885	25.6245	18.9491	17.1889
29	-3.1314	17.3898	16.4356	10.7800	16.8238	8.8145
30	-3.1342	11.2663	7.6318	14.6153	14.7415	22.8869
31	-9.3860	9.3886	6.4083	8.5082	16.3222	21.0484
32	-3.6603	4.9830	2.8064	1.1137	13.4206	18.2560
33	7.7139	-4.0655	-4.3152	-4.8666	8.2651	17.8535
34	12.3849	-3.6795	-4.0429	0.4631	7.3581	19.1173
35	12.3849	10.6401	7.7076	15.7265	11.1939	22.1835
36	16.3025	15.3987	11.8818	19.1908	13.3064	22.1096
37	26.0684	15.4937	11.6490	19.0482	16.4251	23.0228
38	28.8469	24.1293	20.1337	23.1239	19.3182	19.3396

39	25.4232	23.2893	19.6018	20.9574	20.1864	18.1825
40	25.4256	12.8675	9.9740	9.6738	17.9464	18.3284
41	21.1532	6.7746	5.1795	1.3943	14.0213	16.1717
42	15.1488	0.8846	1.4750	-5.8793	8.1696	8.2580
43	10.1402	-3.6463	-2.1706	0.2154	5.0142	7.9702
44	7.6924	-8.5643	-7.4039	0.6007	3.8941	13.7414
45	-3.6629	0.2552	0.1029	-4.8800	3.9901	13.3109
46	-9.3847	-8.6134	-7.3815	-0.2065	5.1096	16.8757
47	0.3189	-1.1602	-0.5428	8.1540	3.7956	16.0512
48	0.3179	5.3205	4.3264	12.5611	4.9489	17.8398
49	-9.3849	22.5808	19.7030	24.8517	12.0026	17.9146
50	-3.6576	20.7625	18.7187	23.4580	7.3322	15.2644
51	7.7114	7.5483	6.9037	14.8648	3.5662	15.3998
52	10.1444	6.0910	6.2836	12.2566	0.3907	12.1584
53	15.1552	-1.3774	-1.0236	8.1651	2.4424	12.7487
54	21.1502	-7.9660	-5.7314	0.9005	0.5932	6.1043
55	18.6335	0.5242	0.6570	-3.2696	1.3257	5.4708
56	4.8804	-7.9021	-5.7025	-3.0422	-0.0118	1.2245
57	3.2662	-3.8167	-2.5604	-5.9103	0.7994	1.9351
58	-3.1344	11.3273	9.2539	7.3087	16.8236	16.2238
59	-3.1379	2.5879	3.7796	-2.2578	8.1730	6.1519
60	3.2647	7.2819	4.3404	12.0043	12.4607	22.9303
61	3.2377	7.4188	10.8221	3.0864	8.9057	-0.0169
62	4.8599	7.8528	12.4168	4.0477	5.0309	-1.1285
63	3.2382	6.3100	9.5787	7.7075	-0.5587	2.2264
64	-3.1233	5.7282	7.7555	7.7161	0.3425	1.9687
65	-3.1267	4.8588	5.8712	4.0529	10.0791	1.4019
66	-9.3597	5.4407	7.0772	3.0853	12.9652	0.9235
67	-3.6980	8.7252	12.1671	3.5225	10.8887	-1.7824
68	7.6727	9.9980	14.0211	0.2010	8.3351	-0.0129
69	10.1037	9.1350	13.9864	4.3506	4.7512	-1.1287
70	15.1264	9.9854	15.4799	7.8552	-0.7118	2.2126
71	21.1605	7.6152	10.4173	10.0787	-3.5982	5.4296
72	18.5425	6.7914	6.9573	10.0872	-3.5216	7.8599
73	21.1635	4.8817	4.8089	10.7593	2.8031	13.6795
74	15.1194	4.3172	3.3462	7.8206	9.2248	13.6538
75	10.1003	4.3269	4.1391	4.3264	13.2064	5.6114
76	7.6560	4.8998	4.6809	0.2064	17.1607	6.2541
77	-3.6980	4.8912	4.4956	3.4990	16.4732	2.2825
78	-9.3592	8.0114	7.2540	2.1663	21.2570	1.7754
79	0.3242	8.0007	8.9258	2.1617	19.4987	2.9552
80	0.3232	11.9889	15.2577	0.5953	11.1747	0.9282
81	-9.3608	11.5362	13.8102	5.4416	15.2842	2.2690
82	-3.6967	8.6107	9.3342	1.9558	20.8739	1.7775
83	7.6748	8.6744	7.6337	3.2262	23.4553	6.4554
84	12.3524	0.7310	0.8810	1.9510	23.7580	6.4515
85	12.3518	0.4735	0.2490	5.4275	24.4883	12.8248

86	16.2760	6.8488	4.3610	0.6020	22.3475	13.1779
87	26.0954	5.7220	2.8544	6.1723	21.0357	16.6320
88	28.9442	4.8566	2.8536	7.4430	11.0002	17.1439
89	25.4400	0.4788	6.5418	12.2205	0.4493	7.7042
90	25.4423	8.7016	4.5054	16.8387	10.0997	22.7961
91	28.9403	7.9852	7.2886	17.4188	0.3462	20.3323
92	26.0802	8.6005	8.9400	17.4162	-0.5570	17.6665
93	16.2605	11.5404	12.1678	16.8465	5.0534	17.6033
94	12.3207	11.9751	10.8397	16.4357	8.9343	19.2420
95	12.3210	9.1046	5.8926	14.7791	16.4936	22.5315
96	7.6565	7.8221	4.1522	13.3322	17.1674	22.5338
97	-3.7050	7.4063	4.6837	14.4323	13.2121	22.9984
98	-9.3603	5.4247	4.3800	12.1138	9.2239	19.2435
99	-3.1199	4.8768	0.2403	12.6461	2.7999	17.6140
100	-3.1237	7.9581	0.8734	16.5938	-3.5252	17.6598
101	3.2505	8.6228	9.3462	16.5821	-3.5993	15.3448
102	10.1110	8.3100	13.8033	12.6571	-2.8754	7.7039
103	15.1383	10.8864	15.2588	12.1178	-0.7132	7.8521
104	16.2773	11.9953	14.0134	14.4237	4.7536	13.6654
105	26.0935	12.9973	13.9965	13.3304	8.3411	13.6209
106	28.9437	12.5653	12.4444	14.7749	10.9061	17.1185
107	30.5856	11.0500	9.5943	11.1315	19.5109	16.6179
108	30.5845	9.9836	7.7780	11.1372	21.2706	18.5257
109	28.9332	6.3193	3.3430	7.8421	22.3461	18.5263
110	26.0780	7.6641	4.7841	6.6871	24.4864	16.1850
111	16.2615	8.4248	6.9357	8.8941	23.7595	16.1795
112	15.1316	8.3402	6.5298	6.7457	23.4560	12.8207
113	10.1083	10.9010	10.4237	8.8865	20.8758	13.1654
114	3.2516	11.9798	13.4742	6.6890	15.2818	6.2378
115	4.8841	13.0067	15.4816	7.8489	11.1759	5.5872
116	18.5719	12.5758	16.1664	6.2093	8.6783	1.3853
117	21.1802	11.0427	16.1637	7.4736	-0.2096	1.9679
118	25.4455	0.7398	7.6887	16.0947	0.4496	15.3447
119	25.4479	8.3940	13.4691	10.7627	-2.8735	5.4354
120	21.1831	9.9823	7.0951	16.4409	12.9966	23.0031
121	7.9273	10.3831	12.3187	14.8567	15.1750	0.0132
122	8.0733	13.1043	13.9846	12.5496	18.2044	-1.0025
123	7.9277	6.1310	6.2512	15.7211	17.3338	2.1336
124	7.2074	-1.3970	-1.0067	19.2454	13.2481	1.9387
125	7.2052	-7.9943	-5.6928	24.9134	5.9071	1.2318
126	8.7139	-1.9268	0.3469	23.4870	5.8004	0.8778
127	7.8651	14.7841	16.7084	12.2348	15.6062	-1.6003
128	8.2720	18.7095	19.9777	8.1504	19.0343	0.0508
129	8.0777	18.0681	18.7016	8.1453	20.8562	-1.0384
130	8.0666	23.3079	21.7171	-0.2259	24.9915	3.3928
131	7.9411	7.5557	6.8870	11.9920	18.9903	6.1538
132	8.0697	-1.1606	-0.5201	19.0827	12.7493	7.9727

133	7.9415	-8.6329	-7.3528	23.1962	2.7635	13.7448
134	8.0628	0.2441	0.1074	25.7172	-2.4239	13.3179
135	8.0738	0.5110	0.6722	26.8356	-0.2128	5.4779
136	8.2559	-7.9300	-5.6709	26.6031	0.3766	6.1080
137	7.8591	-2.3298	0.0965	24.5107	3.1906	2.1438
138	8.7149	8.1066	10.9241	18.4130	4.7571	1.3261
139	0.6126	12.9194	15.5589	14.4256	8.2132	2.2319
140	0.6121	26.6998	26.9075	0.8897	19.4371	0.8256
141	8.7117	25.8653	26.3727	1.0656	15.8535	1.2390
142	7.8628	16.1205	18.3881	11.2838	7.9756	0.6011
143	8.2750	14.0493	16.2322	13.0966	4.4388	5.1887
144	8.5259	9.2844	11.6506	17.4885	1.8579	5.6513
145	8.5252	1.6846	3.5310	21.8142	-3.2325	12.1631
146	8.2704	-3.8242	-2.5426	24.6655	-4.6764	12.7529
147	7.8756	-3.6678	-2.1482	24.1786	0.0456	16.0641
148	8.7883	-8.5871	-7.3826	24.8476	0.7510	16.8857
149	7.2319	5.3515	4.2975	14.6076	16.6961	8.2676
150	7.2359	9.4270	6.4045	10.7653	5.9225	22.8318
151	8.7882	11.3190	7.6177	7.2878	13.2524	21.0401
152	7.8677	15.5426	11.6090	1.4215	17.3446	18.3255
153	8.2543	24.1366	20.1246	-5.8478	18.2190	18.1422
154	8.4951	23.2905	19.5934	-2.2652	15.1997	19.2839
155	8.4955	11.3647	9.2347	10.1655	3.2055	22.0619
156	8.2586	6.7891	5.1673	14.1366	0.3771	22.1591
157	7.8561	5.0130	2.7924	14.9111	-0.2078	22.9103
158	8.7135	-4.0391	-4.3241	21.9675	-2.4250	19.1409
159	7.2096	-3.6473	-4.0502	20.9421	2.7603	17.8625
160	7.2058	7.3331	4.3246	9.6880	12.7512	18.2525
161	7.9426	15.4396	11.8366	1.1398	18.9880	16.2063
162	8.0886	22.5665	19.6910	-4.8352	24.5680	8.8262
163	8.0775	26.2858	23.3859	0.6112	24.9853	9.0685
164	8.2734	27.5073	23.9199	0.2195	20.8654	14.3679
165	7.8729	28.6185	25.5085	-5.8995	19.0442	13.7533
166	8.7866	26.0997	23.0531	-3.0134	15.6295	17.1540
167	0.6039	21.9815	20.6809	2.9968	8.2199	15.8542
168	0.6039	17.3818	16.4494	6.8724	4.7618	17.8732
169	8.7860	0.8912	1.4836	20.7494	-4.6787	17.8459
170	7.8653	2.5902	3.7950	19.3034	-3.2320	15.4090
171	8.2573	15.1591	15.1635	9.5936	1.8567	15.2589
172	8.0736	18.2033	18.5431	7.8031	4.4411	11.7712
173	8.0847	22.5540	22.1536	3.9528	7.9738	12.2569
174	7.9434	27.7772	27.3897	-0.5100	15.8587	5.3327
175	8.0995	29.7970	28.6292	-3.0305	19.4361	5.4796
176	8.0956	29.1053	28.2734	-3.2662	21.3828	1.8067
177	7.9564	26.7957	24.8104	-4.8501	25.4309	3.4589
178	7.2324	10.7118	7.6500	8.4824	16.6920	16.1845
179	7.2374	20.8272	18.6575	0.4375	24.5707	6.4954

180	7.9572	12.9055	9.9428	7.1358	5.8258	22.9494

Table S12. Atomic contributions to the mean polarizability of C_{60} and isomeric structures C_{120}

№	Distributed polarizability of atoms (a.u.)			
	C_{60}	$(C_{60})_2$	Peanut C_{120}	Fullerene C_{120}
1	9.0447	11.7673	19.7919	13.1433
2	9.0492	12.3958	19.7884	13.1278
3	9.0447	17.3076	18.3656	6.8822
4	9.0453	18.7578	17.2407	5.8264
5	9.0384	17.0624	18.3658	5.8176
6	9.0342	14.7038	14.6122	5.5425
7	9.0441	8.7390	11.1645	5.5505
8	9.0526	7.6481	19.7919	5.5402
9	9.0310	10.2283	19.7945	5.8168
10	9.0402	10.2292	14.6194	6.9031
11	9.0450	17.3118	18.3543	6.8941
12	9.0582	19.6617	14.6038	13.1400
13	9.0351	19.6618	11.8477	15.9271
14	9.0405	18.7562	9.6514	15.9165
15	9.0330	17.0630	9.6454	13.1662
16	9.0291	14.7087	11.8529	5.8327
17	9.0252	13.3544	14.6171	5.5614
18	9.0254	6.3288	18.3686	6.9035
19	9.0239	5.3743	19.7960	15.9557
20	9.0200	1.9515	19.7949	17.7890
21	9.0247	-0.2713	17.2278	15.9422
22	9.0228	1.8508	11.8319	15.9456
23	9.0213	1.8556	9.1772	6.8844
24	9.0531	5.3745	6.5552	5.5494
25	9.0596	8.7312	11.8582	5.8195
26	9.0478	11.7568	18.3632	13.1416
27	9.0410	12.3741	18.3576	15.9157
28	9.0333	17.2975	17.2401	17.7189
29	9.0347	18.7585	6.5513	13.1355
30	9.0217	11.7481	9.1903	5.8214
31	9.0167	14.6884	2.8359	5.5428
32	9.0185	13.3287	2.4351	6.8919
33	9.0155	6.3011	2.8304	15.9165
34	9.0287	5.3584	2.4357	6.8909
35	9.0263	7.6289	9.6185	5.5459
36	9.0276	10.1973	11.1360	5.8198
37	9.0327	12.3777	14.5944	13.1337
38	9.0371	17.2982	14.5946	6.8875
39	9.0530	18.7574	11.1553	5.5539

40	9.0368	17.0563	9.6486	5.8241
41	9.0471	14.6854	9.6252	13.1463
42	9.0562	11.7510	6.5427	15.9439
43	9.0471	8.7280	6.5488	15.9445
44	9.0526	5.3603	2.4394	13.1325
45	9.0381	1.8505	1.5365	6.8857
46	9.0511	1.8535	1.5360	5.5492
47	9.0481	-0.2668	1.5344	5.8247
48	9.0274	1.9482	1.5375	13.1430
49	9.0192	10.1977	2.4324	6.8996
50	9.0251	7.6313	6.5401	5.5472
51	9.0305	1.9458	9.6241	5.8238
52	9.0281	-0.2703	6.5453	13.1351
53	9.0191	0.4365	11.8332	5.8262
54	9.0179	0.4363	11.8292	5.5488
55	9.0287	-0.2689	9.1700	6.9005
56	9.0261	1.9532	2.8258	15.9128
57	9.0280	7.6454	2.4322	15.9143
58	9.0223	17.0533	1.5355	13.1563
59	9.0234	12.3948	1.5369	5.8231
60	9.0378	8.7263	2.4340	5.5536
61		18.8077	19.8859	6.8931
62		19.7034	19.8832	15.9546
63		19.7036	18.3889	15.9571
64		18.8076	17.2278	13.1482
65		17.0830	18.3999	6.8996
66		17.0810	14.6297	5.8265
67		17.3118	11.1282	5.5529
68		17.3121	19.8842	5.8250
69		18.8123	19.8843	13.1546
70		17.0799	14.6102	5.5501
71		18.8144	18.3981	6.9087
72		17.3237	14.6308	17.7221
73		12.3933	11.8706	17.7872
74		11.7513	9.6431	17.7880
75		14.7188	9.6247	13.1467
76		13.3234	11.8376	5.8310
77		14.7148	14.6245	5.5493
78		11.7434	18.3916	6.9067
79		12.3692	19.8808	15.9311
80		12.3712	19.8810	15.9354
81		10.1832	17.2420	13.1680
82		10.1851	11.8666	5.8267
83		7.6163	9.1531	5.5587
84		8.7245	6.5372	6.9019

85		5.3635	11.8434	15.9584
86		6.3257	18.4006	15.9620
87		5.3627	18.3866	6.8951
88		8.7251	17.2121	5.5556
89		17.3261	6.5320	5.8257
90		1.9395	9.1322	13.1615
91		7.6331	2.8371	6.9050
92		8.7192	2.4267	5.5507
93		5.3474	2.8453	5.8277
94		1.8503	2.4333	13.1507
95		0.4442	9.6452	17.7958
96		-0.2786	11.1485	17.7234
97		1.9365	14.6364	15.9654
98		7.6346	14.6208	6.9065
99		10.2123	11.1203	5.5635
100		10.2155	9.6261	5.8340
101		11.7362	9.6252	13.1707
102		14.6975	6.5311	13.1561
103		13.2918	6.5324	5.8326
104		6.3008	2.4289	5.5546
105		5.3485	1.5245	6.9121
106		1.8534	1.5275	20.5151
107		-0.2771	1.5272	20.5077
108		0.4443	1.5249	20.5146
109		1.8540	2.4275	20.5174
110		1.8547	6.5423	17.7221
111		-0.2785	9.6557	15.9315
112		1.9323	6.5440	17.7266
113		1.9355	11.8666	15.9296
114		7.6164	11.8434	20.4543
115		8.7138	9.1451	20.4582
116		11.7291	2.8436	20.4641
117		14.6992	2.4298	20.4616
118		12.3893	1.5260	20.4587
119		17.0776	1.5252	20.5158
120		-0.2780	2.4300	17.7954
